# Morphological Configuration of Sensory Biomedical Receptors Based on Structures Integrated by Electric Circuits and Utilizing Magnetic-Responsive Hybrid Fluid (HF)

**DOI:** 10.3390/s22249952

**Published:** 2022-12-16

**Authors:** Kunio Shimada

**Affiliations:** Faculty of Symbiotic Systems Sciences, Fukushima University, 1 Kanayagawa, Fukushima 960-1296, Japan; shimadakun@sss.fukushima-u.ac.jp; Tel.: +81-24-548-5214

**Keywords:** five senses, cutaneous receptors, mimesis, rubber, electrolytic polymerization, hybrid fluid (HF), robotics, magnetic cluster

## Abstract

Biomedical receptors such as cutaneous receptors or intelligent cells with tactile, auditory, gustatory, and olfactory sensations function in the five senses of the human body. Investigations focusing on the configuration of such receptors are useful in the fields of robotics and sensors in the food industry, among others, which involve artificial organs or sensory machines. In the present study, we aimed to produce the receptors for four senses (excepting vision) by morphologically mimicking virtual human ones. The mimicked receptors were categorized into eight types of configured structure. Our proposed magnetic-responsive hybrid fluid (HF) in elastic and soft rubber and proposed electrolytic polymerization technique gave the solidified HF rubber electric characteristics of piezoelectricity and piezo-capacity, among others. On the basis of these electric characteristics, the mimicked receptors were configured in various types of electric circuits. Through experimental estimation of mechanical force, vibration, thermal, auditory, gustatory, and olfactory responses of each receptor, the optimum function of each was specified by comparison with the actual sensations of the receptors. The effect of hairs fabricated in the receptors was also clarified to viably reproduce the distinctive functions of these sensations.

## 1. Introduction

Robotics technology has long been evolving to address the challenges in the areas of industry, surgery, space, and others, with developments such as remote and autonomous driving [1,2]. For implementation, performance requires intelligence similar to the sensory systems of humans. Their peculiarity is that the sensors must also have flexible and stretchable properties. One of the best shortcuts to accomplishing the development of emerging sensory systems is to mimic the biological organism of the living human body. The ideal embodiment of a sensory system would be the fabrication of an artificial receptor having the reactions of the five senses [3,4,5] or of a biosensor [6].

Artificial receptors able to mimic tactile sensations on the skin have been addressed as e-skin [7,8], using strain gauges [9] and matrix networks [10]. In addition, artificial receptors able to mimic auditory [11,12], equilibrium [13,14], gustatory [15,16,17], and olfactory sensations [18,19,20] have been investigated. However, these artificial receptors do not have specific morphological fabrications compared to realistic human receptors, and are often formed by integration with electrical and mechanical substrates that involve electric circuits or chemical responsive systems with bulky structures such as electronic machinery. Therefore, more miniature receptors that simulate the morphology of actual human receptors are needed.

To produce a realistic fabrication, in the present study we use a morphological approach that mimics four of the five human senses, functioning to detect sensations to the human skin, ear, tongue, and nose (we note that the sensation of the eye was not mimicked here because the mechanisms of vision using receptors is too complicated to present comprehensively; it will be fabricated in another report). In this paper, we describe the development of state-of-the-art fabrication of the mechanoreceptors and sensory cells in the tactile, audible, gustatory, and olfactory senses made of soft and elastic rubber by introducing various types of electric circuits and our proposed electrolytic polymerization.

It has already been clarified by anatomical investigation that each of the five senses has typical mechanoreceptors or sensory receptor cells able to sense individually. For example, the tactile sense operates by mechanoreceptors such as Ruffini endings, which are characterized by a low-frequency vibrating mechanical response. However, if we could develop new types of sensors beyond those already known by estimating the characteristics of the overall mimicked receptors and cells of the five senses, they would be useful for improving artificial sensors. Therefore, our purpose is to detect the optimal morphological configuration of mimicked mechanoreceptors for all senses. As we experimentally investigate the typical properties of mechanical force, vibration, thermal, auditory, gustatory, and olfactory responses of each mechanoreceptor [21] by comparison with the results of actual cutaneous receptors, we specify the optimum structure for each sensation.

## 2. Materials

### 2.1. Five Senses and Receptors

**Figure 1:**
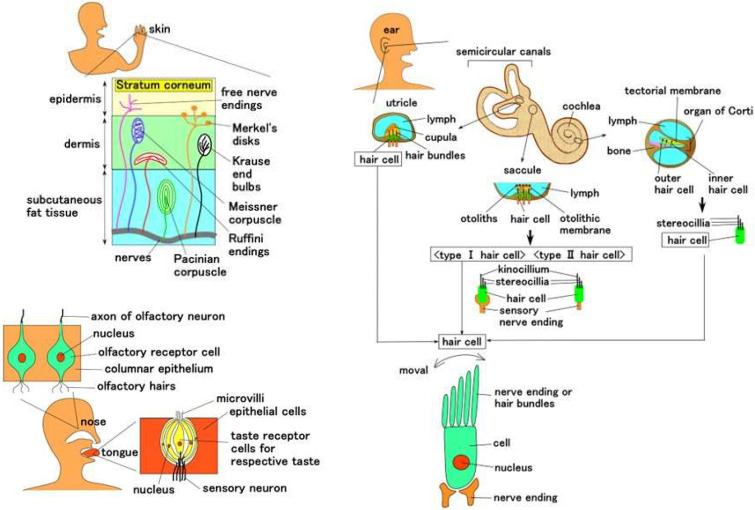
Mechanoreceptors and sensational cells in human five senses except for visible sensation.

The mechanoreceptors and sensory cells in the human skin, ear, tongue, and nose have functions of tactile, auditory, gustatory, and olfactory sensations, respectively, as shown in Figure 1. We did not deal with the receptors in the visible sense because the photovoltaic mechanism is too complicated to present them easily and elliptically. In a special case for the human skin, the mechanoreceptors are recognized to be specified as cutaneous receptors: free nerve endings [22], Merkel’s disks [23], Krause end bulbs [24], Meissner corpuscles [25], Ruffini endings [26], and Pacinian corpuscles [27]. These tactile receptors are feasible to demonstrate the sensory systems. Although the auditory, gustatory, and olfactory hair cells are not categorized in the mechanoreceptors at the field of biomedical dissection, their configurations can be thought to be formed in a structured body, such as tactile receptors. For example, the auditory hair cell and the olfactory receptor cell can form like a ball, while the gustatory hair cell forms like a ball with a layer that has structures like an onion, as shown in Figure 1.

**Figure 2:**
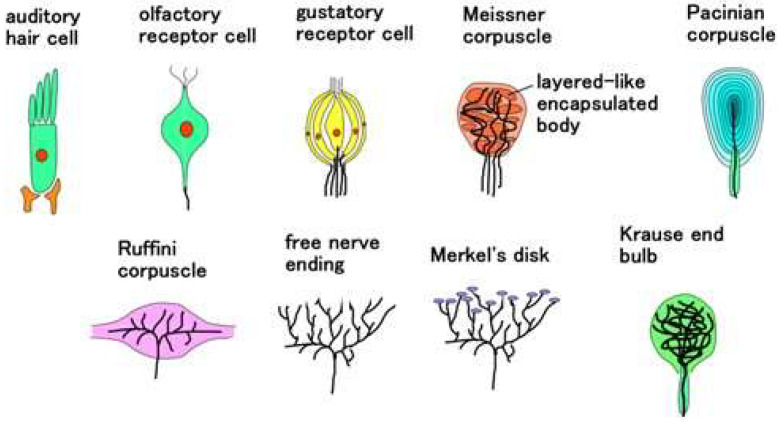
Comprehensively summarized morphology of receptors and hair cells in five senses except for visible sensation.

The receptors and hair cells are comprehensively summarized, as illustrated in Figure 2. Morphologically, Meissner’s corpuscles have a few encapsulated bodies, which are joined together. Pacinian corpuscles form many layered tissues. Ruffini corpuscles form longitudinally which involves the longitudinally elongated nerves. Free nerve endings and Merkel’s disk have widely spread nerves, and the latter form a lump in the tip of the nerve. The Krause end bulb forms like a ball, in which nerves are mixed through crosslinking and coil form. On the other hand, as for auditory and olfactory cells, it can be considered that they form an encapsulated shell. As for gustatory cells, they can be considered to have plural encapsulated shells which are respectively sensitive to the variegated tastes. In each encapsulated shell, biochemical reactions occur and can be transferred to the nerves. Therefore, we can suppose that the structure has encapsulated shells in which the nerves, including Ruffini corpuscles and Krause end bulbs, are not wired.

From these configurations of the receptors and cells, the sensations can be mimicked by the morphological structure configurated by the electric circuit as the nerve functions.

### 2.2. HF Rubber

**Figure 3:**
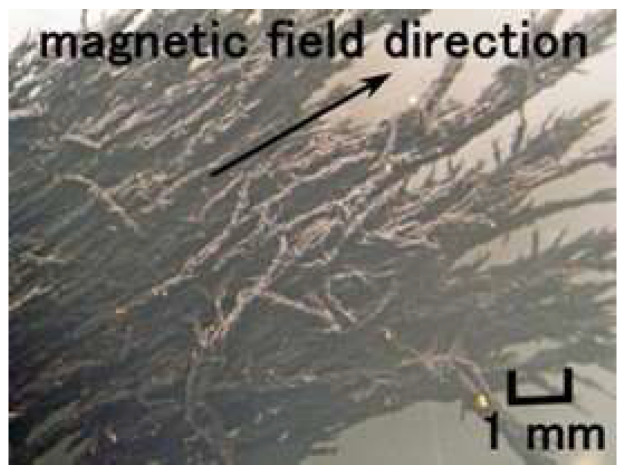
Magnetic clusters aligned along the applied magnetic field in HF.

The prerequisite condition to morphologically mimic these receptors is to utilize a soft and elastic rubber, as the human body and skin are very soft and elastic. Shimada proposed an intelligent fluid suitable for compounding a rubber and investigated the optimization of the constitution and the properties of the rubber compounded with the fluid. At the first stage, our proposed fluid is a magnetic compound fluid (MCF), which is a magnetically responsive colloidal fluid that consists of 1-μm-diameter ordered metal particles such as Ni or Fe and 10-nm-diameter sphere magnetite (Fe_3_O_4_) particles coated by an oleic acid surfactant in a solvent, such as water and kerosene [28]. MCF is produced by compounding the magnetic fluid (MF), involving 10 nm Fe_3_O_4_ coated by a surfactant, with the metal powders. At the next stage, for the convenience of not needing MF, a novel magnetically responsive fluid, consisting of Fe_3_O_4_ and Fe metal particles, surfactant, water, kerosene, silicone oil (Q), and polyvinyl alcohol (PVA), has been proposed as a hybrid fluid (HF) [29]. For the purpose of developing a state-of-the art rubber which brings about the viability of merging together with any variegated discrepant rubber, such as natural rubber (NR), chloroprene rubber (CR), Q-rubber, and urethane rubber (U), among others, the solvents are mixtures of water, kerosene, Q and PVA. PVA has a role of emulsion polymerization so that those discrepant solvents can be mixed. MCF and HF have a paramagnetic property. When a magnetic field is applied, many needlelike or rug-like magnetic clusters are created not only in MCF or HF but also in the rubber compounded with MCF or HF, as shown in Figure 3, enhancing the electrical and thermal conductivity. Therefore, MCF and HF rubbers become conductive rubbers, so that they can be piezo-resistive and be utilized for the production of sensory receptors. The foregoing magnetism and magnetic clusters have been presented in detail in our previous studies [29].

We prepared HF rubbers 1–4 with each respective constitution, as presented in Table 1. First, we produced HF with the following components: 3 g water, 3 g kerosene, 3 g silicon oil (KF96 with 1 cSt viscosity, which would solidify Q; Shin-Etsu Chemical Co., Ltd., Tokyo, Japan), 21 g PVA, 3 g Fe_3_O_4_ particle (Fujifilm Wako Chemicals Co., Ltd., Osaka, Japan), 3 g Fe particles (M300, about 50 μm particles; Kyowa Pure Chemical Co., Ltd., Tokyo, Japan), and 4 g sodium hexadecyl sulfate aqueous solution for surfactant. All ingredients were mixed with an agitator under air evacuation. Next, we produced HF rubbers 1 and 4 in a liquid state by mixing. As for HF rubbers 2 and 3, they were solidified with electrolytic polymerization, which has been proposed as an easy solidification technique regardless of diene and non-diene rubbers without using vulcanization by sulfur. In our previous study, the detailed electric conditions have been presented [30]. Here the carbonyl Ni powder (No. 123, Yamaishi Co., Ltd., Noda, Japan) has microns-ordered particle sizes with bumps on the surface. Because TiO_2_ (anatase type, Fujifilm Wako Chemical Co., Ltd., Osaka, Japan) is a typical body for electron transfer, we made the HF rubber more conductive by compounding with TiO_2_. Moreover, regarding HF rubber 2, it involved a liquid by using a vacuum evacuation, because it can be permeable by our proposed electrolytic polymerization technique [31], as is explained in detail in the following section.

### 2.3. Electrolytic Polymerization

The electrolytic polymerization technique for the production of the HF rubber and the artificial receptor may be summarized as follows. It is a state-of-the art engineering technique for the production of artifacts made of rubber.

- solidification
- creation of built-in voltage
- production of porous rubber, and infiltration with a liquid
- adhesion a rubber to metal

As for (a) above, the molecules of the rubber can be crosslinked, which has been presented [32]. This technique is a novel solidification of a rubber in contrast to the ordinary vulcanization with a sulfur.

As for (b) above, the built-in voltage is generated by the electrons and holes created by ionization induced from the particles and molecules of the HF rubber, whose physical mechanisms are presented in the following section regarding an equivalent electric circuit.

As for (c) above, we can make a rubber permeable by the electrolytic polymerization using metallic hydrate. Na_2_WO_4_ 2H_2_O is optimal as the metallic hydrate, as has been presented [31]. As in other engineering applications, the permeable rubber can be a filter made of rubber. Moreover, any liquid can be infiltrated in the permeable rubber by vacuum evacuation so that the rubber can involve any liquid. If we use a battery liquid as the infiltrated liquid, we can produce rubber having the function of battery. In the present study, we used a glycerin which is dielectric. As a result, the permeable and infiltrated rubbers are novel in contrast to the ordinary rubber.

**Figure 4:**
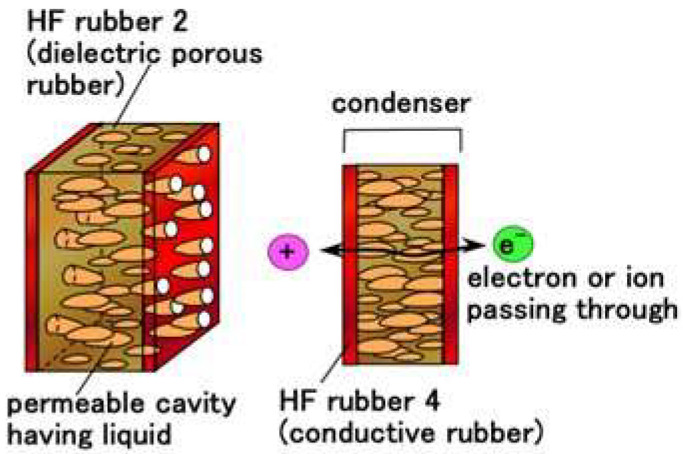
Physical model of the permeable HF rubber 2.

Regarding the structure of HF rubber 2, it has many porous rubber parts produced by this technique. HF rubber 2 can be produced involving any liquids through vacuum evacuation. Therefore, depending on the kind of liquids involved, the HF rubber 2 can be a condenser, a capacitor, and so on. In the present study, we produced the permeable HF rubber 2 involving a glycerin because it becomes a condenser. The ions and electrons then pass through HF rubber 2, as shown in Figure 4.

As for (d) above, we can adhere a rubber to any metals by the electrolytic polymerization with using metallic hydrate. Na_2_WO_4_ 2H_2_O is optimal as the metallic hydrate, which has been presented [33]. This technique is significant for the production of a sensor, because the electric wires adhered to the sensor are electrodes for measuring a voltage that they can cohere on the rubber. Especially, this technique can enable thin electric wires to adhere to the rubber sensor. It was used in the fabrication described below.

### 2.4. Artificial Receptors

#### 2.4.1. Fabrication

**Figure 5:**
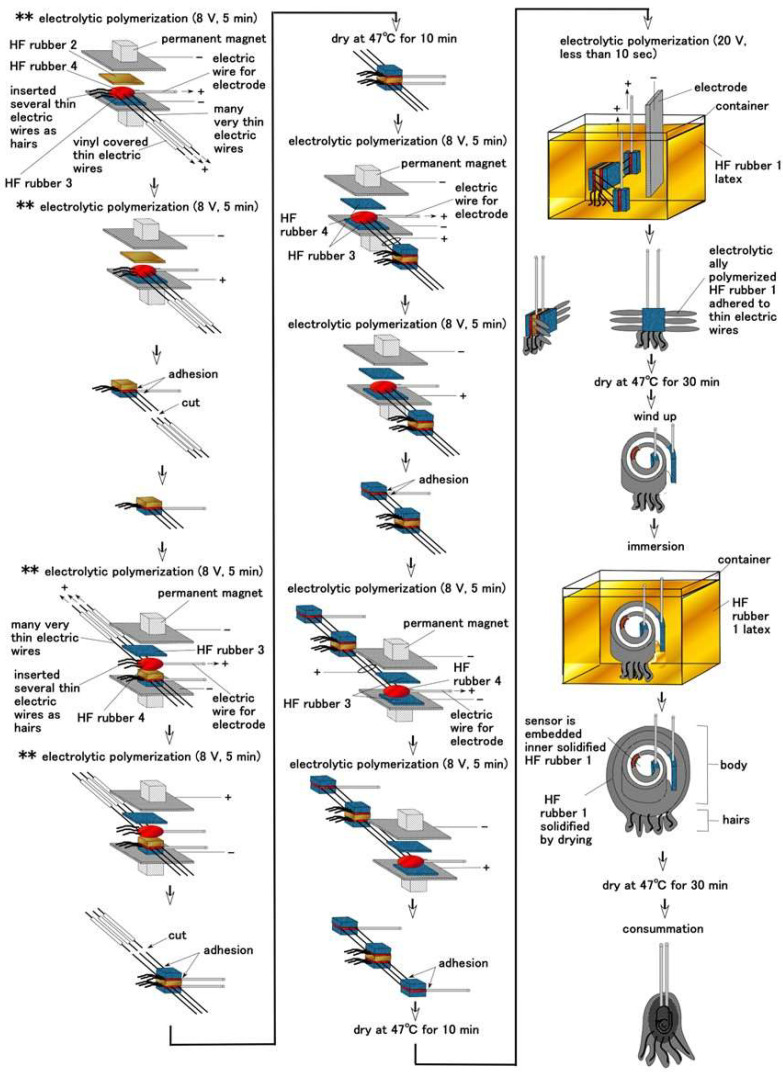
Production process of Meissner’s corpuscles-like sensor: ** as shown in the figure means the production process which ensures that the hairs B do not touch the electric wires for electrodes, and which designates as “non-conductive hair”. This character instructs the same meaning in Figure A1 in Appendix A.

**Figure A1:**
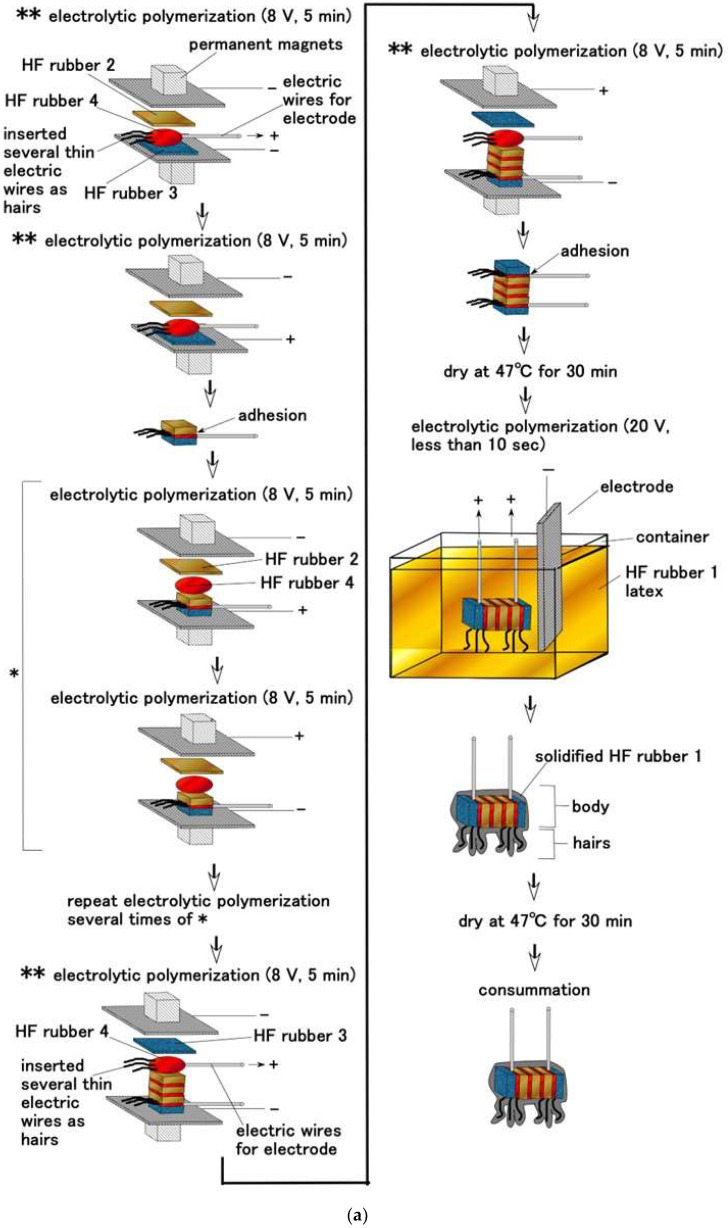

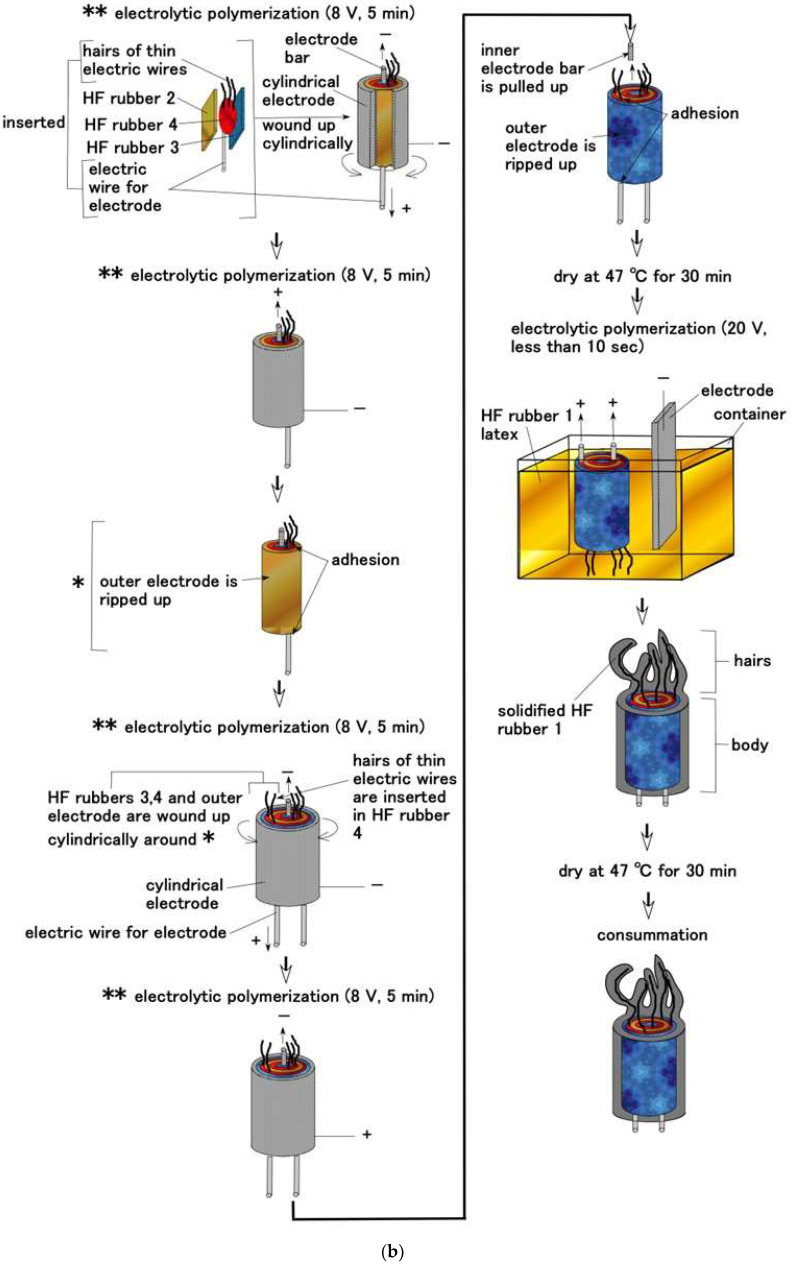

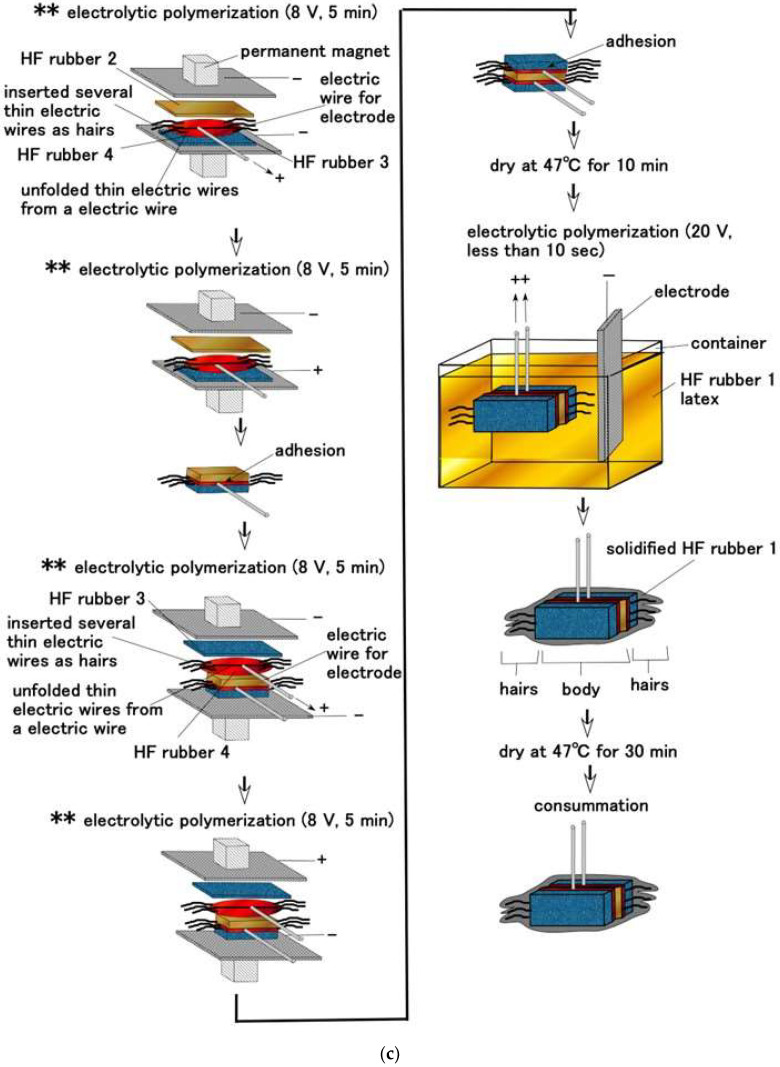

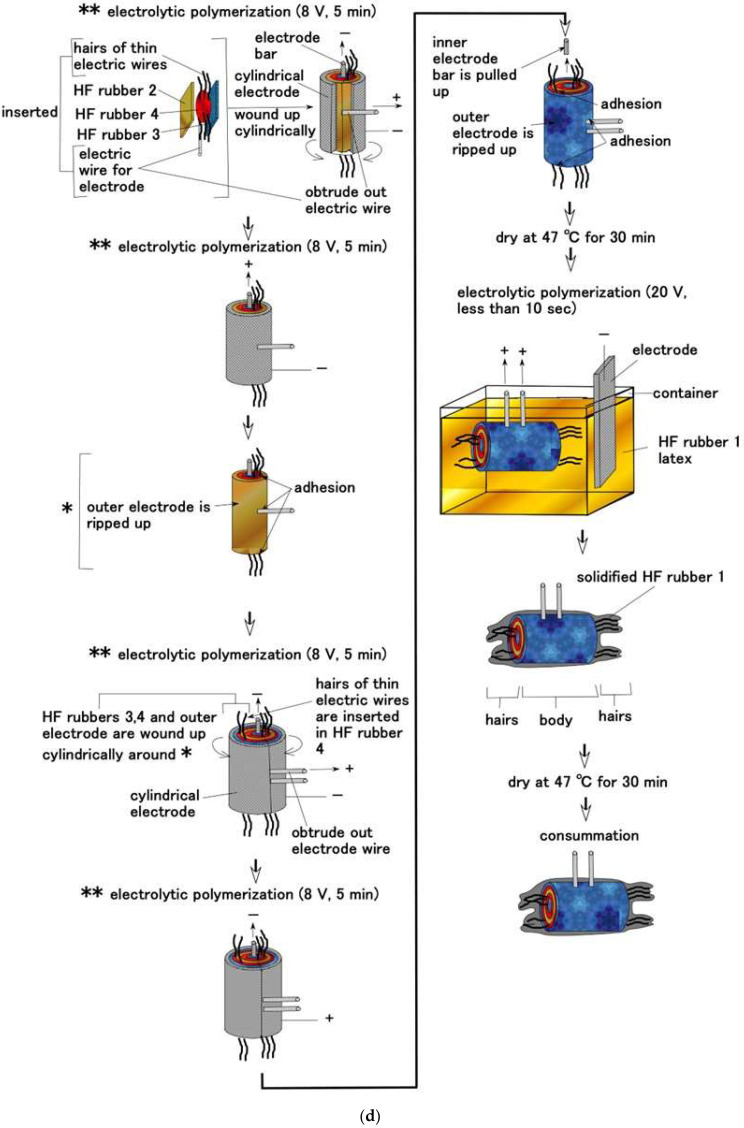

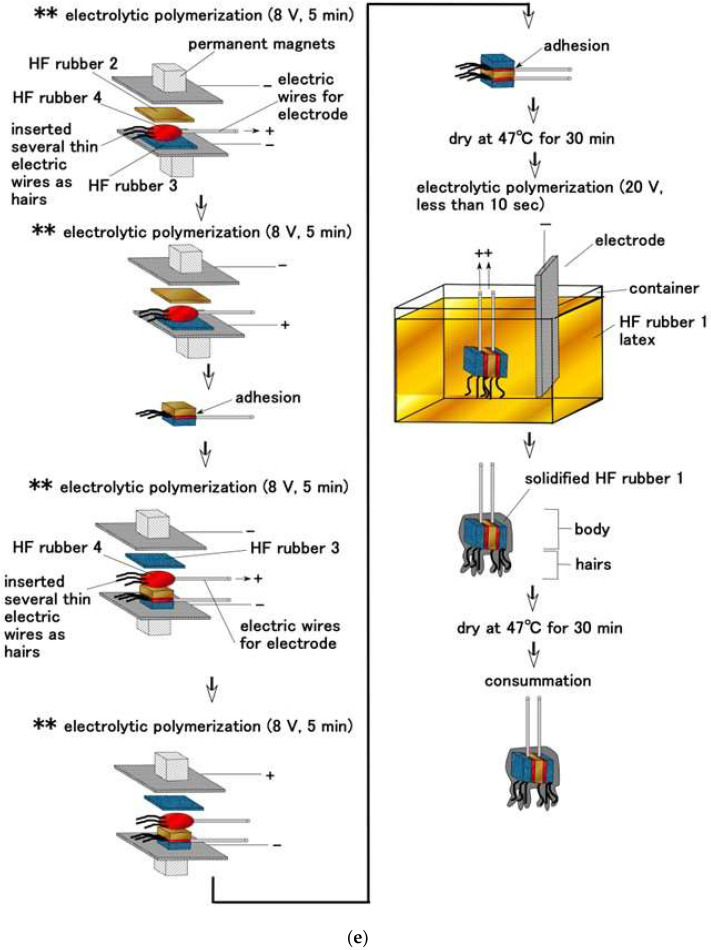

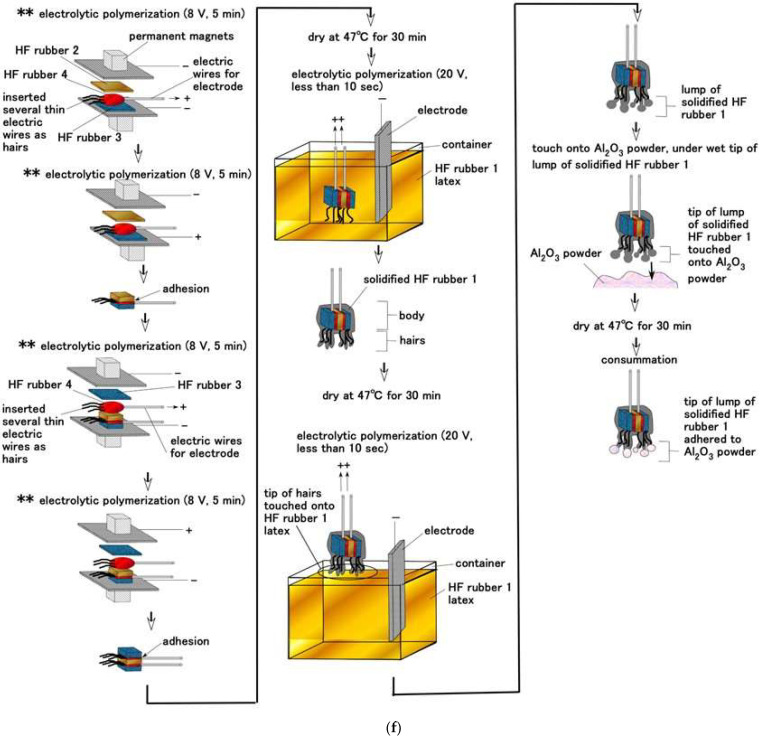

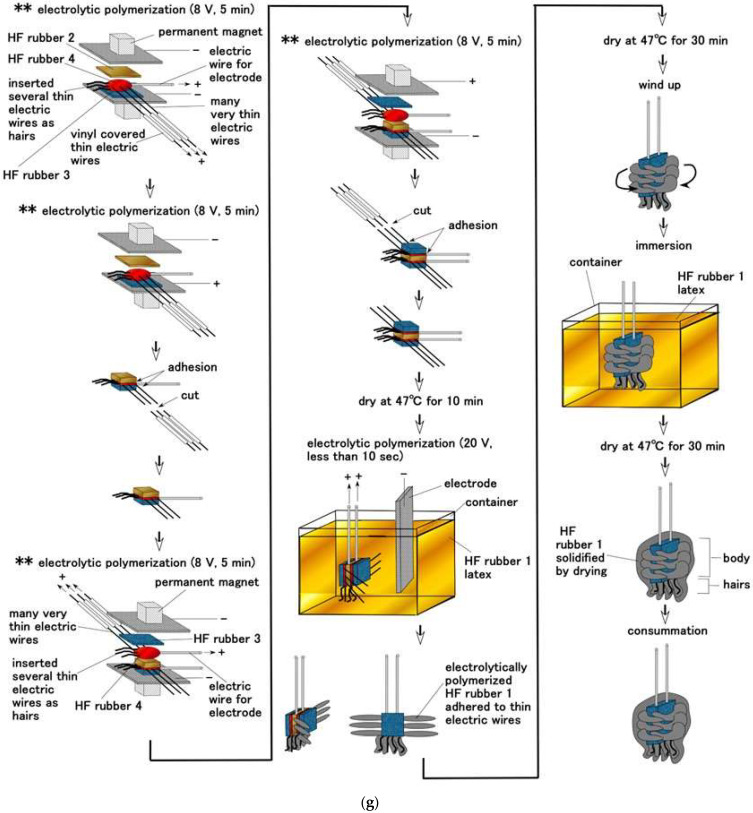
Fabrication process: (**a**) layered Pacinian corpuscles; (**b**) cylindrical Pacinian corpuscles; (**c**) layered Ruffini corpuscles; (**d**) cylindrical Ruffini corpuscles; (**e**) free nerve endings; (**f**); Merkel’s disk; (**g**) Krause end bulbs. * means repeating phase. ** as shown in the figure means the production process which ensures that the hairs B do not touch the electric wires for electrodes, and which designates as “non-conductive hair”.

After HF rubbers 1–4 were prepared, the next phases of fabricating the artificial receptor involve the sensor production, as shown in Figure 5 and Figure A1 in Appendix A. In the present main text here, just one typical process, for a Meissner-type receptor, is presented, because the set of illustrations for all eight variegated production processes for the respective receptors is too long for delineation.

**Figure 6:**
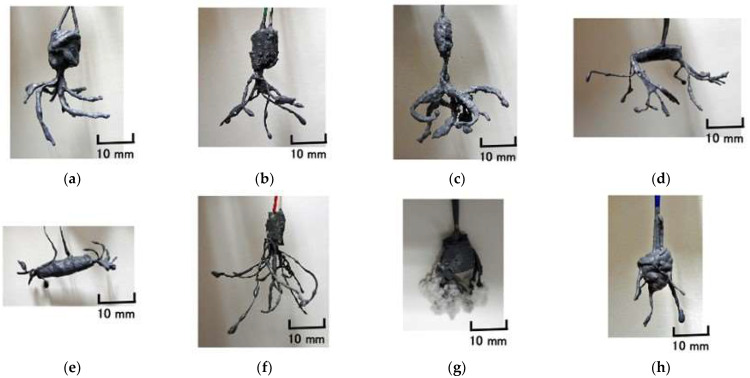
Images of fabricated artificial receptors: (**a**) Meissner corpuscles; (**b**) layered Pacinian corpuscles; (**c**) cylindrical Pacinian corpuscles; (**d**) layered Ruffini corpuscles; (**e**) cylindrical Ruffini corpuscles; (**f**) free nerve endings; (**g**) Merkel’s disk; (**h**) Krause end bulbs.

Figure 6 shows the final HF rubber-based fabricated sensors.

#### 2.4.2. Equivalent Electric Circuit

**Figure 7:**
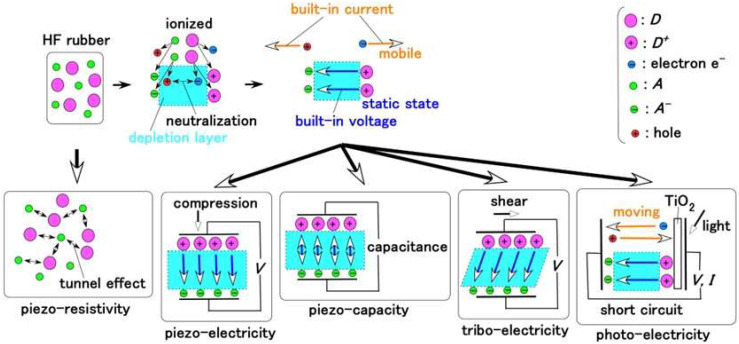
Electric conditions provided by the HF rubber.

Regarding the electric behavior of the HF rubber, by applying an extraneous electric field to the HF rubber, an electron can pass through nonconductive materials of the particles of Fe_3_O_4_, Fe, Ni, and TiO_2_ and molecules of rubber, solvent, and surfactant through the tunnel effect, which has been presented in our previous study [34], and whose condition corresponds to piezo-resistivity, as shown in Figure 7.

On the other hand, the particles and molecules are ionized through electrolytic polymerization on the HF rubber, so that the rubber plays the role as p-type and n-type semiconductors with the configuration of the ionized particles and molecules as acceptor A^−^ (acceptor) and D^+^ (donor); these phenomena have been presented in detail in our previous study [34]. The electrons and holes created by ionization induced from the A and D, are mobile to generate a built-in current, and in the area between them, the electrical situation is regarded to be neutralized so that the area forms a depletion layer. Therefore, the electrical situation of A^−^ and D^+^ is in a static state and occurs in a built-in voltage. Consequently, the condition corresponds to piezoelectricity for the application of compression, to piezo-capacity for electric charge, and to triboelectricity for the application of shearing, as shown in Figure 7, which has been presented in our previous study [35]. These conditions also correspond to a condenser, predominantly. In contrast, the materials with piezoelectricity and piezo-capacity are well-known piezo elements. In addition, a study has also been conducted focusing on other physical sensors with triboelectricity [36].

If light is emitted on the surface of TiO_2_ attached on the HF rubber, the photovoltage and photocurrent are generated as an organic solar cell, whose condition corresponds to photoelectricity, as shown in Figure 7, which has been presented in our previous study [37]. In contrast, material responding photoelectricity is well known as constituting a solar cell, particularly, organic solar cell.

Consequently, MCF and HF rubbers can simultaneously have the overall foregoing electric conditions as shown in Figure 7. Although HF rubbers 1–4 provide the overall electric conditions, they each have a particular role as follows: HF rubber 1 involves a wire of the hair which is connected with HF rubber 2; HF rubber 2 has the role of condenser or capacitor as shown in Figure 4; HF rubber 3 has the role of outer cover of the receptor, and HF rubber 4 is adhesive. In contrast, other artificial haptic sensors such as e-skin [38] and physical sensor [39] play functions on the basis of a particular electric condition as shown in Figure 7 for their respective engineering and biomedical applications.

**Figure 8:**
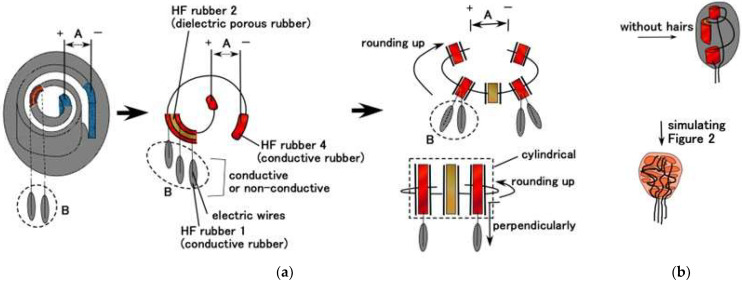

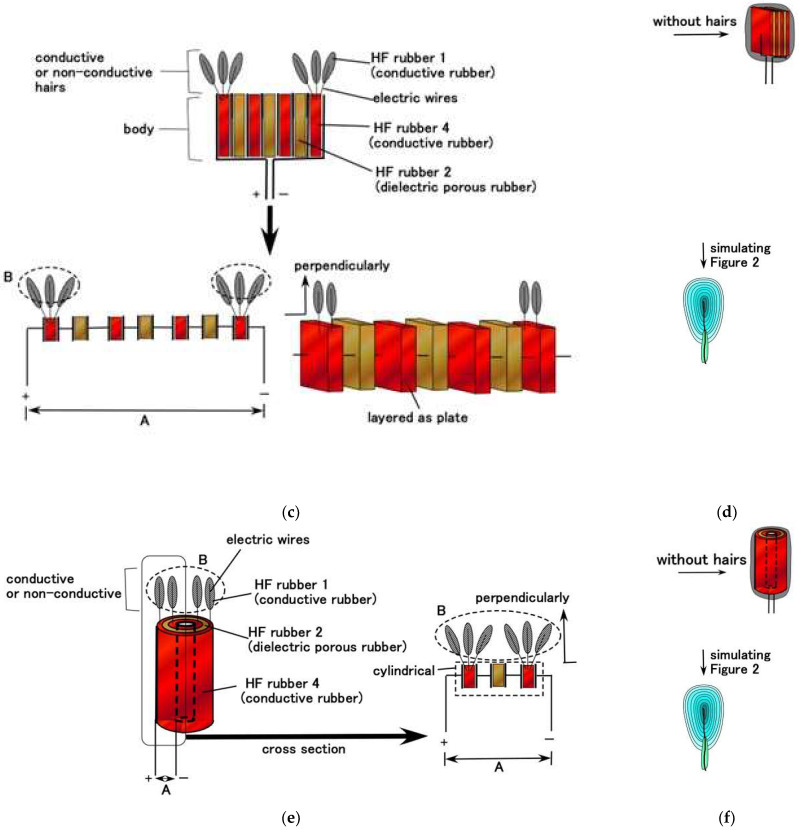

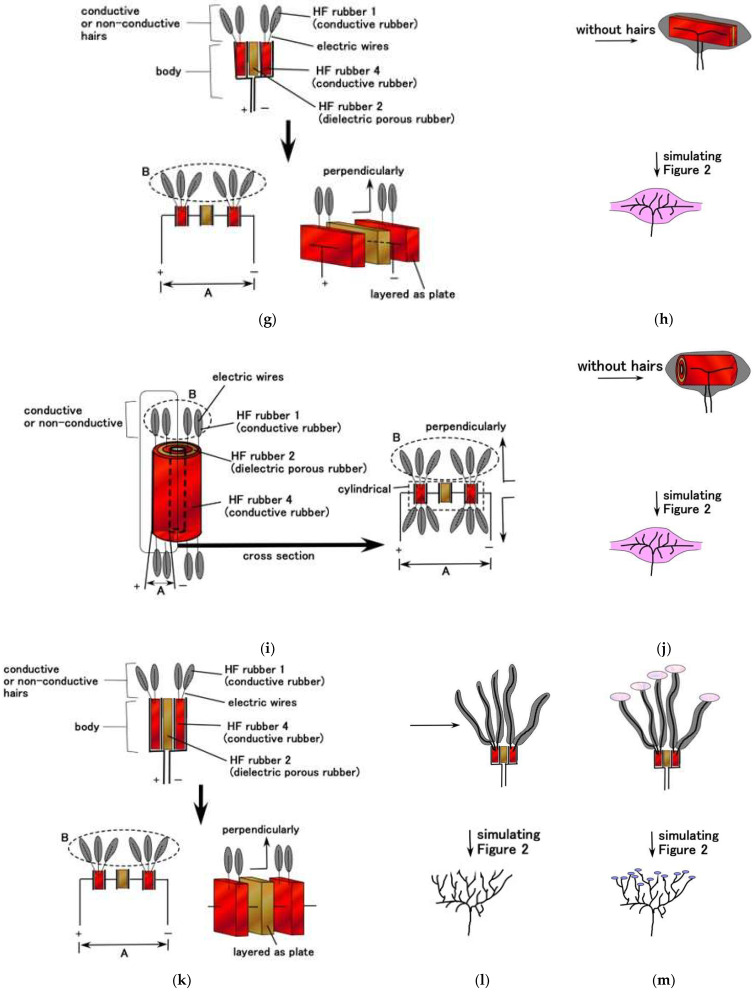

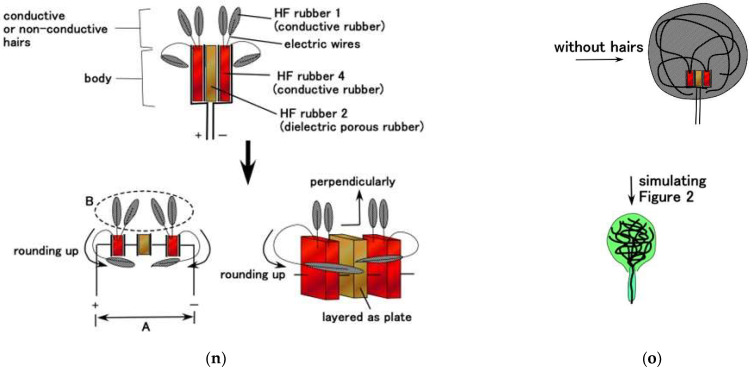
Equivalent electric circuit induced by the fabrication process and configuration compared to the actual structure: (**a**,**b**) Meissner corpuscles; (**c**,**d**) layered Pacinian corpuscles; (**e**,**f**) cylindrical Pacinian corpuscles; (**g**,**h**) layered Ruffini corpuscles; (**i**,**j**) cylindrical Ruffini corpuscles; (**k**,**l**) free nerve endings; (**m**) Merkel’s disk; (**n**,**o**) Krause end bulbs. (**a**,**c**,**e**,**g**,**i**,**k**,**n**) equivalent electric circuit; (**b**,**d**,**f**,**h**,**j**,**l**,**m**,**o**) configuration compared to the actual structure.

The equivalent electric circuit can be configured in the artificial receptor, as shown in Figure 8. We can consequently presume the parallels as contrasting the practical morphological receptors as shown in Figure 2 by inducing from the equivalent electric circuit.

Regarding hairs attached to the receptor, we can produce three types of device: with conductive hairs, without hairs, and with nonconductive hairs. The first corresponds to the production as shown in Figure 5 and Figure A1 in Appendix A. The second and the third are two other types. The receptor without hairs delineated as B “no hair”. The second receptor B is delineated as B “non-conductive hair” (the production process ensures that the hairs B do not touch the electric wires for electrodes as shown ** in Figure 5 and Figure A1 in Appendix A). In contrast, the case where the hairs B are conductive (the production process ensures that the hairs B touch on the electric wires for electrodes) is called “conductive hair”.

By estimating the equivalent electric circuit induced by the fabrication process, as shown Figure 5 and Figure A1 in Appendix A, the configuration of the receptor can result in the actual structures, as shown in Figure 2 and Figure 8. The critical point is that the electric circuit provides the morphological version (hereafter also ‘fabrication’ or ‘device’) of the receptor and that the variegated types of the electric circuit induce the variegated morphological types of the receptor.

As for Meissner corpuscles, when the hairs B are not used, the condensers made of HF rubbers 2 and 4 are wound, as shown in Figure 8a, and come down to, as shown in Figure 8b. The device squares with the type of Meissner corpuscles, as shown in Figure 2. In the same way, in case of the finalized fabrication without hairs B, the devices, as shown in Figure 8d,f,h,j,o, square with the layered Pacinian corpuscles, cylindrical Pacinian corpuscle, layered Ruffini corpuscles, cylindrical Ruffini corpuscles, and Krause end bulbs, as shown in Figure 2, respectively. As for the free nerve endings and Merkel’s disk, the finalized fabrication with hairs B, as shown in Figure 8l,m, squares with the ones shown in Figure 2, respectively. Thus, taking into account the electric circuit, the production process of our fabricated receptors effectively provides the configuration of the realistic receptors as shown in Figure 2.

On the other hand, for auditory and olfactory cells, when we consider how to substitute a capsule single condenser, their configurations can be considered as having a condenser with hairs, such as those seen in Figure 8l,m of the configurations of free nerve endings and Merkel’s disk. As for the gustatory cell, it can be considered to have plural capsules with hairs, in contrast to the auditory and olfactory cells structured by a single capsule. Namely, the gustatory cell has plural condensers which are structured in the configurations of free nerve endings and Merkel’s disk as shown in Figure 8l,m. The configuration corresponds to the one of layered Pacinian corpuscles as shown in Figure 8c,d in case of having hairs.

## 3. Experimental Procedure

In general, the mechanoreceptor has mechanical and chemical properties as a response for extraneous stimuli from the external environment such as touching, vibration, heating, cooling, hearing, and odor, among others. The properties have been investigated, and the characteristics have been clarified in several categories: force, vibration, temperature, sound, smell, and so on [40,41,42,43]. The present experiment was conducted utilizing the classification of extraneous stimuli in terms of the following several responses.

**Figure A2:**
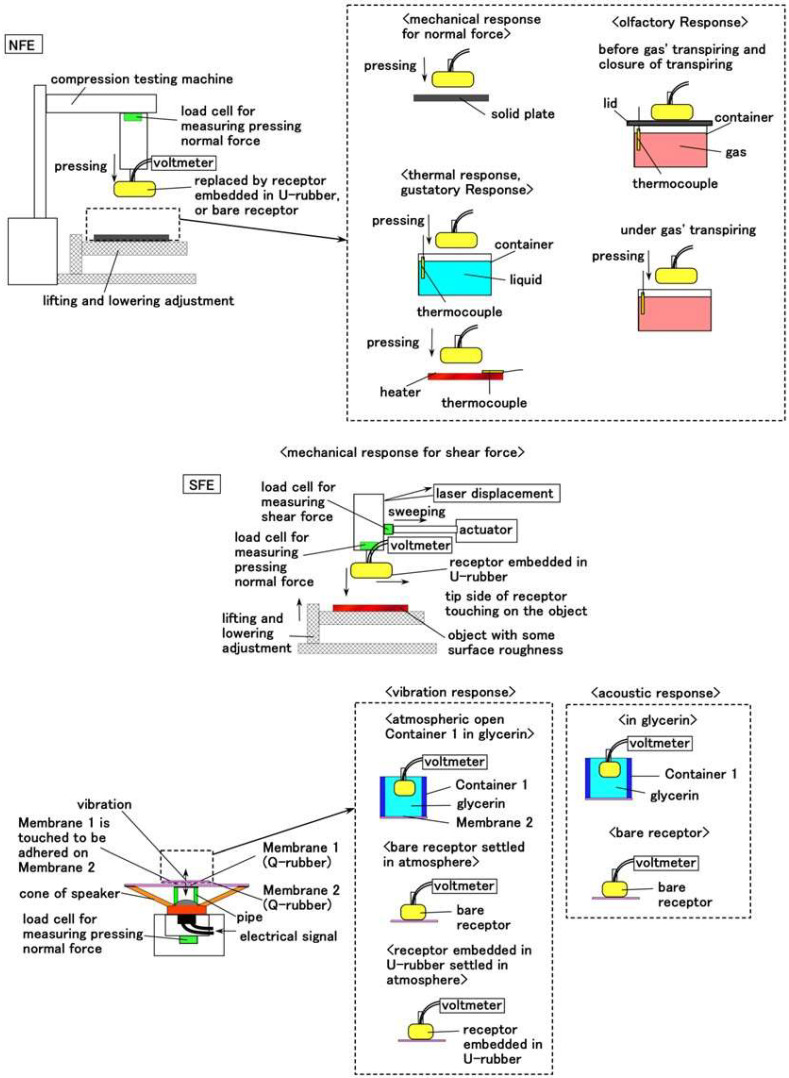
Schematic diagram of experimental apparatus for the five senses except for the visible sense.

The detailed experimental procedure has been already enunciated [28,31,44,45]. Therefore, the outline is presented here by referring to Figure A2 in Appendix B.

### 3.1. Mechanical Response

We addressed two types of the application of normal and shear forces on the receptor fabricated in the previous section. The receptor was embedded in the mimicked human thumb made of U-rubber, whose production technique has been presented in our previous study [31] and is adapted here. The receptor was recumbent in the thumb with an 8-mm-diameter rigid acrylic resin bar mimicking a human bone [45]. The surface was coated with the solidified NR- and CR-mixed rubber, simulating a human skin such as stratum corneum or epidermis. We dealt with the situation of applying normal and shear forces on the thumb, because the actual reality is that the receptor is embedded in a human body and that when a force is applied, the receptor responds via the tissue.

Regarding normal force, a 40-mm-diameter rigid acrylic resin cylinder pushes down the mimicked thumb set on a rigid plate at a velocity of 100 mm/min using a compression testing machine (SL-6002; IMADA-SS Co., Ltd., Toyohashi, Japan) and then moves up. The motion is repeated a few times. The measurement system has been presented in our previous study as a normal force experiment (NFE) apparatus [28,44] and was adapted here. The voltage from the receptor in the thumb was measured using a voltage meter (PC710, Sanwa Electric Instrument Co., Ltd., Tokyo, Japan).

Regarding shear force, the thumb which was installed on an actuator was swept on an object with some surface roughness at a sweeping speed of 5 mm/s, sweeping distance of 50 mm, and pressing force of around 0.02 N. The measurement system has been presented in our previous study as shear force experiment (SFE) apparatus [44] and was adapted here. The voltage from the receptor was measured by the voltage meter (PC710). The surface roughness was #60 (*R_a_* = 30.91 μm, *R_q_* = 36.38 μm, *R_y_* = 139.4 μm), #80 (*R_a_* = 24.11 μm, *R_q_* = 28.6 μm, *R_y_* = 103.9 μm), and #100 (*R_a_* =16.01 μm, *R_q_* =19.56 μm, *R_y_* =84.5 μm) alternatively on a smooth acrylic resin surface with *R_a_* = 0.03 μm, *R_q_* = 0.03 μm, and *R_y_* = 0.2 μm.

### 3.2. Thermal Response

The thermal experiment was conducted in two types of thermal bodies, namely, heater and water. The thumb in which the receptor is attached on the compression testing machine (SL-6002) was pushed on the heater or water poured in a container. The thumb was the same one used in the mechanical response experiment. The measurement system has been presented in our previous study, using an NFE apparatus [44], and was adapted here. The voltage from the receptor was measured by a voltage meter (PC710).

### 3.3. Vibration Response

Each of the bare receptors which was not embedded in a thumb made of U-rubber was settled in the atmosphere and attached to a soft membrane. In addition to the experiment with the bare receptor, we settled the receptor inserted in a container with glycerin. The container was attached on the soft membrane. Moreover, we also used the receptor embedded on the thumb made of U-rubber and attached on the soft membrane. The soft membrane, which exhibited 3619 GPa Young’s modulus and wa made of Q-rubber, was adhered to the edge of an acrylic resin cylinder attached on the cone of the speaker. The vibration was applied on the soft membrane by the speaker. The measurement system has been presented in our previous study [44] and was adapted here. The voltage from the receptor was measured using a voltage meter (PC710). The container, i.e., Container 1, had 65-mm outer diameter and 57-mm inner diameter. Container 1 stored with glycerin since it is optimal in acoustic sensation, as shown in our previous study, which indicates that the experimental structure is relevant to the actual acoustic response for artificial cochlea [44].

### 3.4. Acoustic Response

Using the same experimental apparatus foregoing in the vibration response, a sound was applied on the bare receptor settled in the atmosphere and the Container 1 with glycerin as well as the bare receptor exposed to the atmosphere, respectively. The measurement system has been presented in our previous study, and the voltage from the receptor was measured by a voltage meter (PC710) [44].

### 3.5. Gustatory Response

On basis of the situation that the taste sensation is obtained by the hairs of the gustatory receptors in a tongue, we dunked the bare receptor in a liquid with a flavor, and the voltage from the receptor was measured by a voltage meter (PC710). In the experiment of gustatory as well as olfactory response, just a bare receptor was used. The measurement system has been presented in our previous study [45]. We used liquids having several concentrations with five taste sensations, namely, sweetness (a sugar solution in water), saltiness (a salt solution in water), sourness (a rice vinegar solution in water), bitterness (a familiar coffee solution in water), and umami (a favorite Japanese food tuna powder solution in water). An ORP instrument (YK-23RP-ADV, SatoTech Co. Ltd., Kanagawa, Japan) was used to measure the redox potential (ORP), while a pH meter (PH-208, SatoTech Co. Ltd., Kanagawa, Japan) was used to measure the pH of these liquids. The receptor attached on the compression testing machine (SL-6002) touched the liquid at a velocity of 100 mm/min using an NFE apparatus. In addition, the cyclic voltammogram plots show the relationship between the electric current *I* and voltage *V* using a potentiostat (HA-151B, Hokuto Denko Co. Ltd., Tokyo, Japan) at 50-mHz scan rates with the potential domain of −1.5–1.5 V, because gustation depends on electrochemical behavior.

### 3.6. Olfactory Response

Although this experiment has not been dealt with in our previous studies yet, we can use the same experimental apparatus as in the foregoing gustatory response. The bare receptor attached on the compression testing machine (SL-6002) was inserted in a container filled with ammonia gas at a velocity of 100 mm/min with using an NFE apparatus. The voltage from the receptor was measured by a voltage meter (PC710). The gas that we used is vapor from an ammonia water with 28 wt% inside a container. The olfactory relation between *I* and *V* by using potentiostat (HA-151B) was also measured.

## 4. Results and Discussion

### 4.1. Mechanical Response

**Figure 9:**
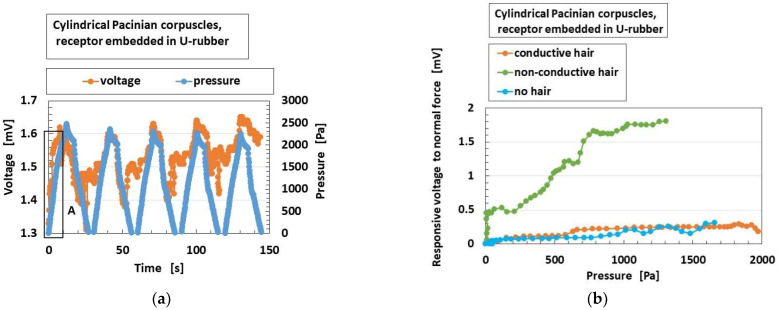
Mechanical response for normal force: (**a**) exemplary response of the built-in voltage to the applied pressure in case without hairs; (**b**) effects of the presence and conductivity of the hairs.

As for the application of the normal force, Figure 9a shows the exemplary response of the built-in voltage to the applied pressure in overall receptors in case without hairs. From the experimental data, we extract the built-in voltage to the pressure at the initial period as A, delineated in Figure 9a, indicating the electric response to the pressure. The parameter is called “responsive voltage to normal force”.

The “responsive voltage to normal force” means the gradient or ratio of the change over of elapsed time to the changing force. The response time is the instantaneous time reactive to the application of the force, and means the transient time through the changes of the applied force. It is defined as (Δ*V*/*V_o_*)/Δ*p*, where Δ*V* is changes of the voltage of the sensors, *V_o_* the initial voltage, and Δ*p* the changes of the applied pressure. As shown in Figure 9a, it is 6.61%/Pa. In contrast, in case of e-skin made of borophene-paper and polyimide-tape as a different type of sensor, Δ*I*/*I_0_*/Δ*p* is 0.0076%/Pa, where *I* is the current of the sensor [46]. Because the changing signal from the sensor to the applied pressure enhances, the HF rubber sensor has a more rapid response time. The cause of the difference is that the borophene-paper and polyimide-tape are solid materials which are not compressible transversely to the normal force on the materials in spite of having a large bending. On the other hand, HF rubber is deformable transversely to the normal force. Therefore, the absolute value of the response time at area A in Figure 9 is rapid, 0.24 ms. In fact, it has already been enunciated that MCF rubber has high sensitivity, which is the same as in the case of HF rubber.

Figure 9b shows the effects of the presence and conductivity of the hairs on this parameter, which is epitomized to be presented because the overall receptors have the same results. The responsiveness of the voltage to normal force is enhanced by the presence of the hairs; however, the conductivity of the hairs is irrelevant. The reason is that the responsiveness of the voltage to the normal force is greater as the area of the reception of the force is wider.

**Figure 10:**
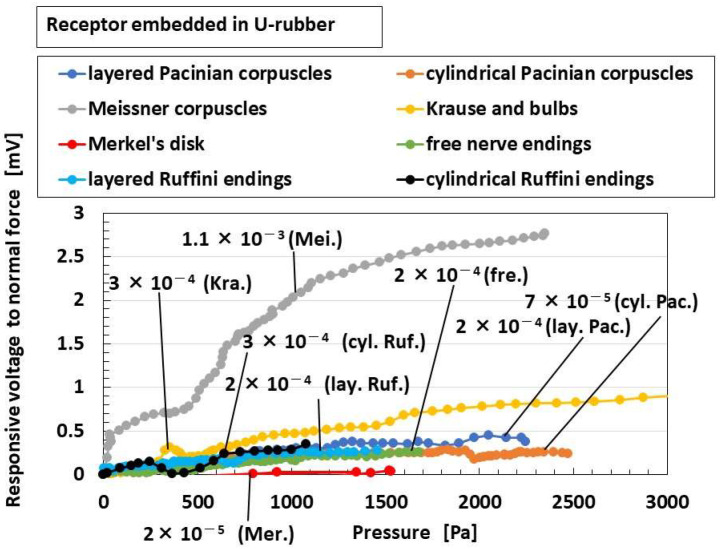
Comparison of responsive voltage to normal force among overall receptors in the cases without hairs: Mei. (Meissner corpuscles), lay. Pac. (layered Pacinian corpuscles), cyl. Pac. (cylindrical Pacinian corpuscles), lay. Ruf. (layered Ruffini corpuscles), cyl. Ruf. (cylindrical Ruffini corpuscles), fre. (free nerve endings), Mer. (Merkel’s disk), Kra. (Krause end bulbs).

Figure 10 shows the comparison of “responsive voltage to normal force” among overall receptors in the case without hairs. Meissner corpuscles are most responsive, followed by Krause end bulbs. The results can be explained as follows: the configuration has longer electric circuit in the receptor, as shown in Figure 2. That is, as the area of the reception of the force is wider, then the responsiveness of the voltage to the force becomes greater.

**Figure 11:**
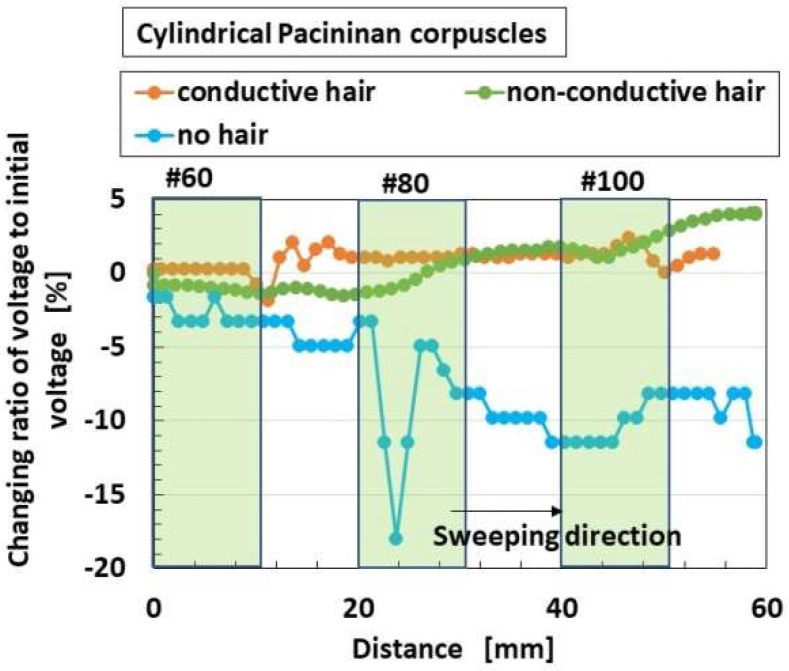
Effects of the presence and conductivity of the hairs on mechanical response for shear force.

**Figure 12:**
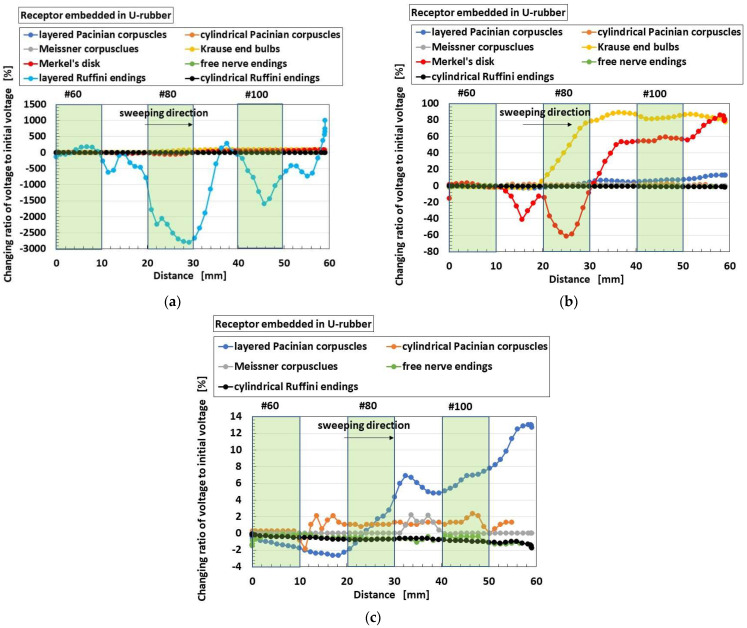
Comparison of responsive voltage to shear force among overall receptors in the case without hairs: (**b**) is magnified from (**a**); (**c**) is magnified from (**b**).

As for the application of the shear force, the changing ratio of the built-in voltage from the initial state before sweeping is arranged. As shown in Figure 11 and Figure 12, the built-in voltage of the HF rubber changes either from positive to negative, or from negative to positive, which is viable on the basis of the following physical mechanism [45]. First of all, as for the position of the static-state ionized particles and molecules as shown in Figure 7, all the particles and molecules are not necessarily aligned face-to-face. There are many pairs of face-to-face particles and molecules, because the constituents are mixed under the application of a magnetic field. It is different from the situation of the ordinary piezo-material as the particles are aligned face-to-face so that the distinct position of the positive and negative electric fields is structured throughout the material. In contrast, regarding HF rubber, there are many parts with face-to-face positive and negative electric field positions, and the summarized electric state can be measured positive or negative by a voltmeter, and can easily change by the ambience around the HF rubber. It is evaluated as an initial built-in voltage before the application of a force. Here we must pay attention that the change of the built-in voltage by the application of a force is more significant to focus on than the initial built-in voltage. By the deformation of the HF rubber under the shear motion, each position of the static-state ionized particles and molecules is changed while adhering on the surrounding rubber so that the summarized electric state also changes such as in Figure 11 and Figure 12. Incidentally, the HF rubber deformed by shearing concentrates to one location, and then the concentrated rubber disperses to be even, whose motion is like the movement of a worm. The cause is due to the very softness of the rubber. The phenomena have been enunciated in our previous paper. In conclusion, the built-in voltage changes are as shown in Figure 11 and Figure 12.

Figure 11 shows the effects of the presence and conductivity of the hairs on this parameter; the overall receptors have the same results as the receptor of Figure 11. The responsiveness of the voltage to shear force is not affected by the non-conductive hair and the conductive hair. The reason is that the responsiveness of the voltage to the shear force is changed all over by the structural effects of morphology.

Figure 12 shows the comparison of responsive voltage to shear force among overall receptors in case without hairs. The results in the case of shear force are different from that of normal force. Layered Ruffini endings are most responsive, followed by Krause end bulbs and Merkel’s disk. From the results, the configuration has a longer electric circuit in the receptor. That is, as the area of the reception of the force is wider, then the responsiveness of the voltage to the shear force becomes greater. As for shear force, the layered-type configuration such as layered Ruffini endings is deformable enough to be responsive, and the branched-out configuration such as Merkel’s disk is wide enough to be responsive.

### 4.2. Thermal Response

**Figure 13:**
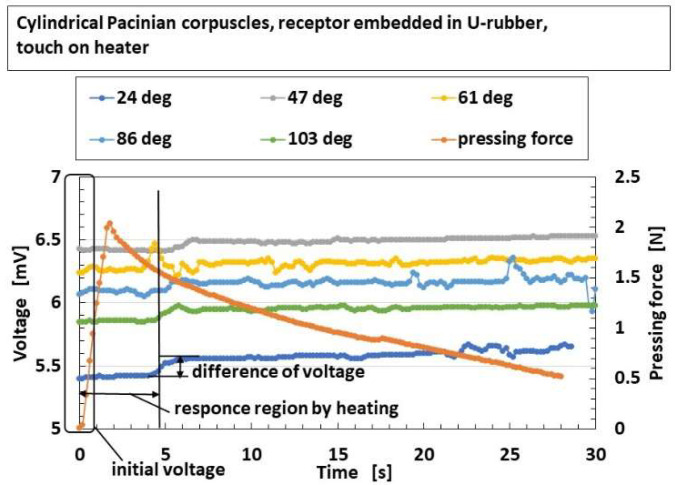
Exemplary response of the built-in voltage to the applied heat in the case without hairs. The degrees designate the temperature of the surface of heater. The force pressed on the heater is also presented in the figure.

As for the application of thermal response, Figure 13 shows the exemplary response of the built-in voltage to the applied heat in overall receptors in the case without hairs. As shown in the figure, the parameter “initial voltage” means the response of the built-in voltage to heat in case of touching a thermal body, i.e., the surface of the heater or the water. The parameter “difference of voltage” means the changes of the built-in voltage inducing the internal thermal energy.

**Figure 14:**
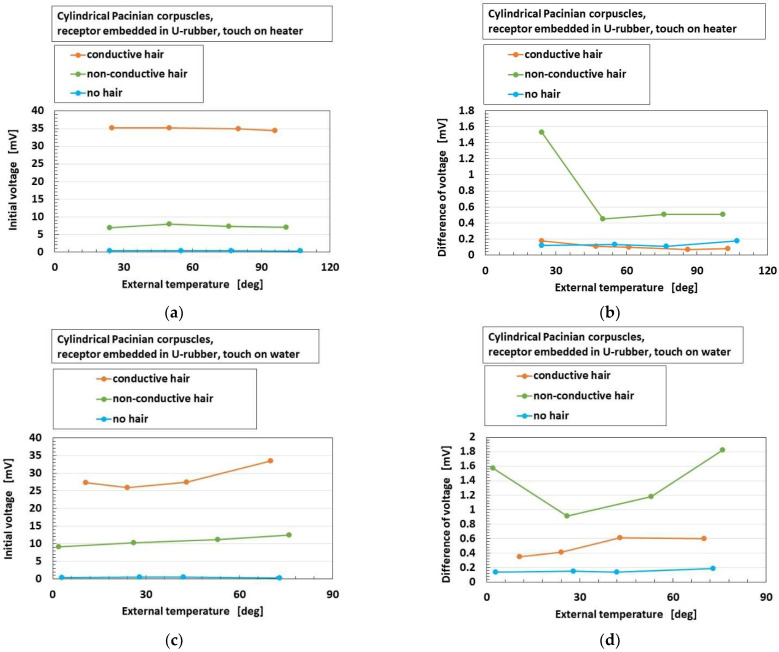
The effects of the presence and conductivity of the hairs on the thermal response: (**a**,**b**) on heater; (**c**,**d**) on water; (**a**,**c**) initial voltage; (**b**,**d**) difference of voltage.

Figure 14 shows the effects of the presence and conductivity of the hairs on these parameters for heater and water, and the overall receptors have the same results. The abscissa “external temperature” means the temperature of the surfaces of heater and water. The responsiveness of “initial voltage” is enhanced by the presence of the hairs; however, the conductivity of the hairs is relevant. The reason is that the responsiveness of the voltage to the heat is enhanced by the presence of the conductive hairs as heat transmits through the hairs. As for “difference of voltage”, the responsiveness of the voltage to heat is enhanced by the presence of the hairs; however, the conductivity of the hairs is irrelevant here. These results are independent of the kind of thermal bodies of heater and water.

**Figure 15:**
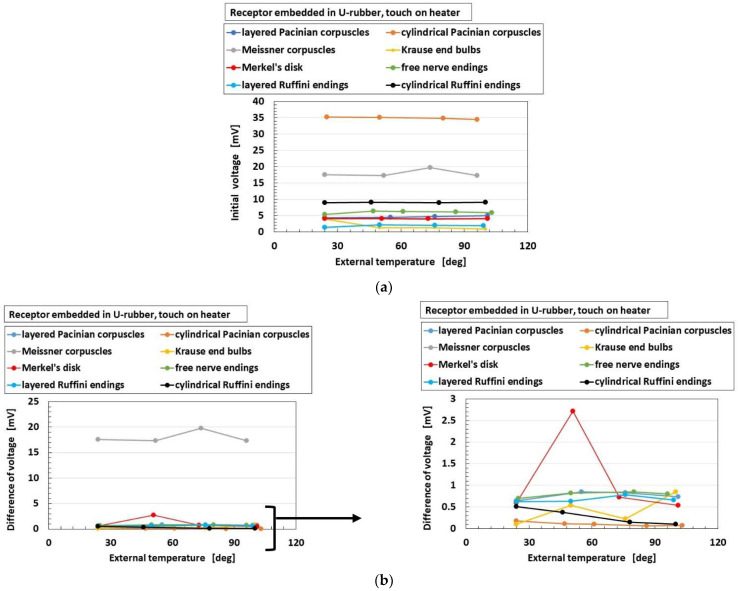
Comparison of responsive voltage to heat among overall receptors as for heater in the case without hairs: (**a**) initial voltage; (**b**) difference of voltage.

**Figure 16:**
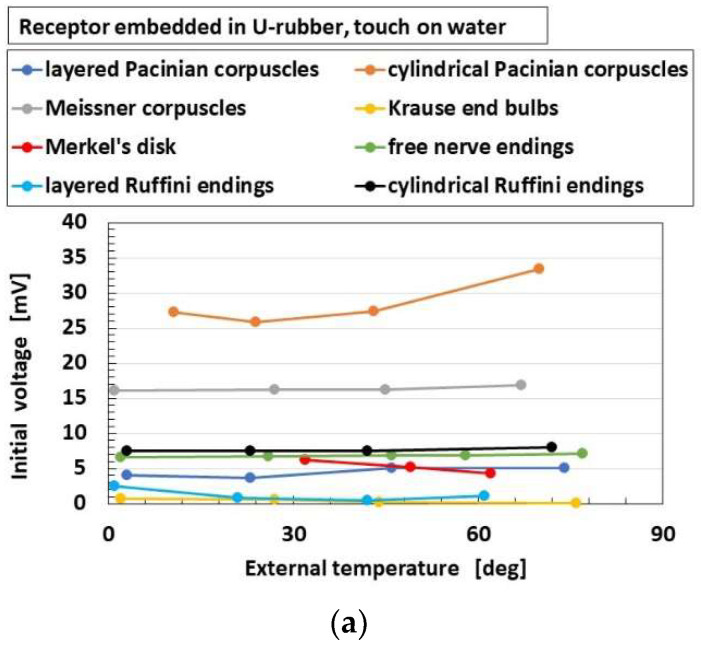

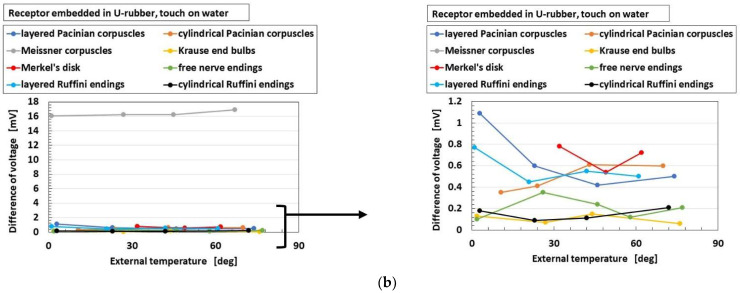
Comparison of responsive voltage to heat among overall receptors as for water in the case without hairs: (**a**) initial voltage; (**b**) difference of voltage.

Figure 15 and Figure 16 show the overall comparison of these parameters to heat among receptors for heater and for water, respectively, in the case without hairs. As for “initial voltage”, cylindrical Pacinian corpuscles are the most responsive, followed by Meissner corpuscles for both heater and water. As for “difference of voltage”, Meissner corpuscles are the most responsive, followed by Merkel’s disk for both heater and water. The results show that the more complicated configuration of electric circuit in the receptor, as shown in Figure 2, is capable of transmitting more heat. These results are independent of the kind of thermal bodies of heater and water.

### 4.3. Vibration Response

**Figure 17:**
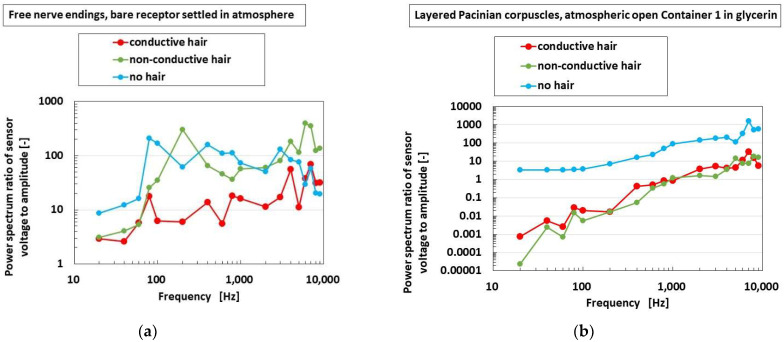
The effects of the presence and conductivity of the hairs on the vibration response: (**a**) bare receptor settled in atmosphere; (**b**) receptor immersed in glycerin in the container with open atmosphere.

The built-in voltage to the applied vibration was analyzed using first Fourier transform (FFT analysis) as the power spectrum. The analyzed parameter was arranged as the ratio of the built-in voltage to the amplitude of the applied vibration. Figure 17 shows the effects of the presence and conductivity of the hairs on the parameters in cases of the bare receptor and the insertion in the container; overall, the cases of receptors have the same results. A larger power spectrum ratio in the abscissa denotes more response to the vibration. The enhancement of the responsiveness of the voltage to the vibration is independent of the presence and the conductivity of the hairs. The reason is that the vibration directly affects the receptor in itself.

### 4.4. Acoustic Response

**Figure 18:**
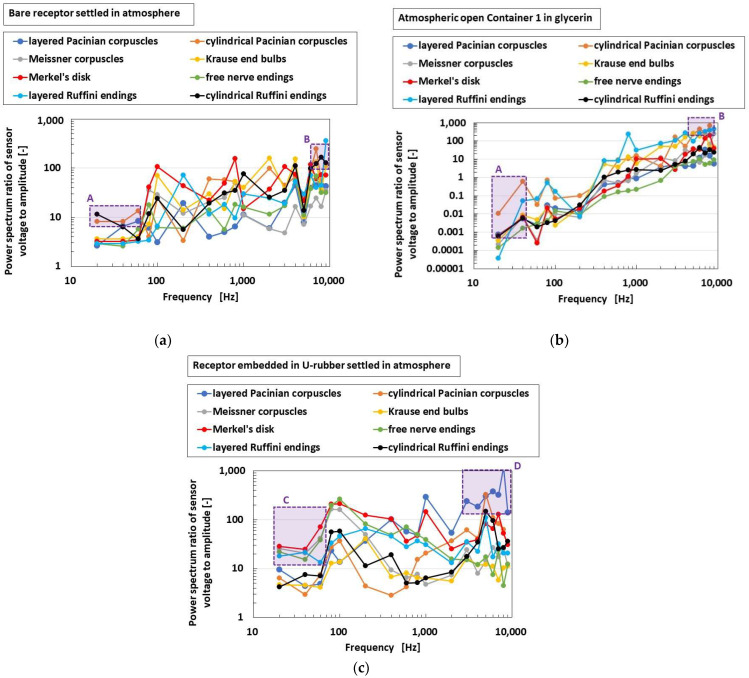
Comparison of responsive voltage to the vibration among overall receptors in case with conductive hairs: (**a**) bare receptor settled in atmosphere; (**b**) receptor immersed in glycerin in the container with open atmosphere; (**c**) receptor embedded in U-rubber settled in atmosphere.

Figure 18 shows the comparison of responsive voltage to the vibration among overall receptors in cases of bare receptor, insertion in the container, and thumb, in the presence of conductive hairs. As for the bare receptor settled in atmosphere and the receptor immersed in glycerin in the container with an open atmosphere, cylindrical Pacinian corpuscles and cylindrical Ruffini endings are the most responsive at low frequency (as indicated by A), followed by Pacinian corpuscles and Krause end bulbs at high frequency (as indicated by B). These results are different from the following case due to the effect of the outer substance of the U-rubber. As for the receptor embedded in a U-rubber settled in atmosphere, Meissner corpuscles, Merkel’s disk, free nerve endings, and layered Ruffini endings are the most responsive at low frequency (as indicated by C), while layered Pacinian corpuscles, cylindrical Pacinian corpuscles, and Merkel’s disk are the most responsive at high frequency (as indicated by D). This case realizes the actual conditions of a human body.

### 4.5. Acoustic Response

**Figure 19:**
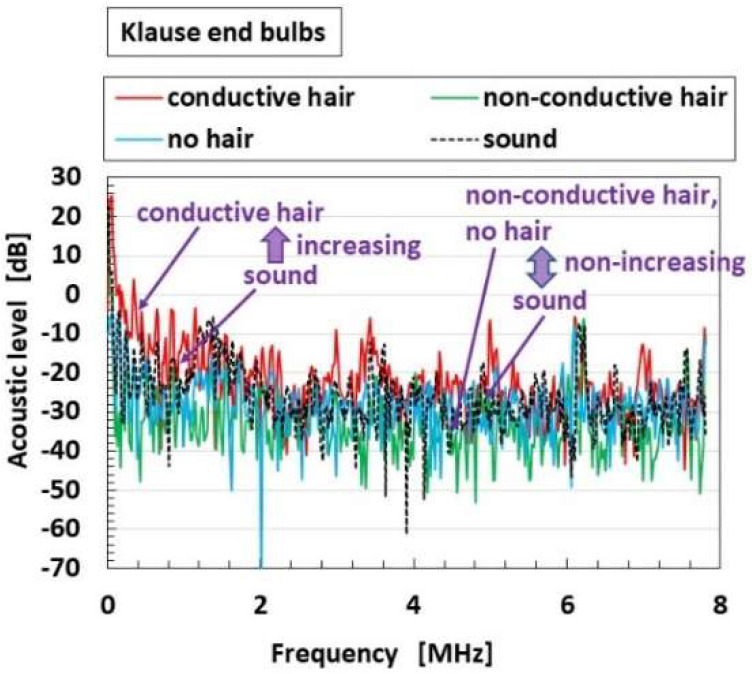
The effects of the presence and conductivity of the hairs on the acoustic response in the case of receptor inserted in the container.

Figure 19 shows the effects of the presence and conductivity of the hairs on the acoustic level induced from the built-in voltage to the applied sound in case of the receptor inserted in the container; the cases of overall receptors have the same results. The acoustic level of sound is based on the applied sound. In the abscissa, a larger acoustic level than that in the case of “sound” denotes more response to the sound. The enhancement of the responsiveness of the built-in voltage to the sound is dependent on the conductive hairs. The reason is that the sound vibration transmitted through the hairs is significant.

**Figure 20:**
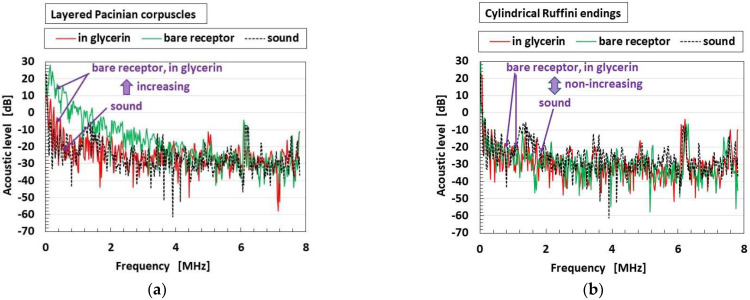
Typical results of responsive voltage to the sound among overall receptors in cases of the bare receptor and the receptor inserted in the container, in the presence of conductive hairs: (**a**) the most responsive; (**b**) not responsive.

Figure 20 shows the comparison of epitomized responsive voltage to the sound culled from overall receptors in cases of the bare receptor and the receptor inserted in the container, for receptors with conductive hairs. Figure 20a is the most responsive case, while Figure 20b shows lack of response. These are typical cases, and other cases are not presented here because there are many results. The ultimate results are that the most responsive to the sound are the layered Pacinian corpuscles and free nerve endings, followed by Meissner corpuscles, Krause end bulbs, and Merkel’s disk.

### 4.6. Olfactory Response

The objective of the present work is to investigate the difference of the sensory properties shown by the variegated receptors, which differentiates this study from the previous works [44,45]. The previous works dealt with just a particular receptor.

**Figure 21:**
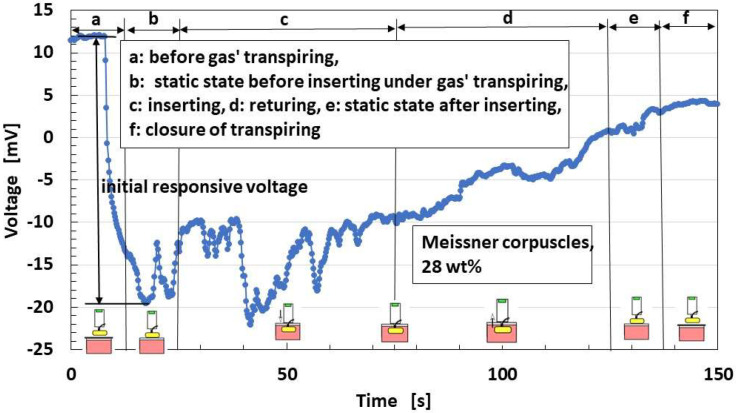
Exemplary response of the built-in voltage to odor in the case with conductive hairs.

The bare receptor is inserted into a container with ammonia water, and examples of the built-in voltage changes in the overall receptors are as shown in Figure 21, in the case with conductive hairs. In the figure, the schematic of the experimental situation is simultaneously delineated. By removing the lid from the container, the built-in voltage changes instantly, and the change is measured as an “initial responsive voltage”.

**Figure 22:**
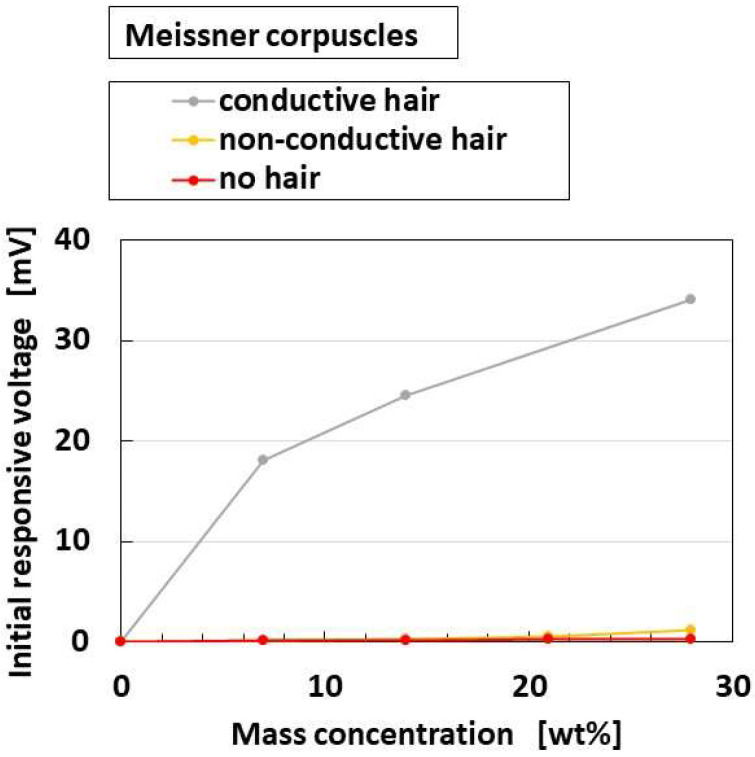
Effects of the presence and conductivity of the hairs on the olfactory response.

Figure 22 shows the effects of the presence and conductivity of the hairs on the parameter “initial responsive voltage”; the overall receptors have the same results. The responsiveness of “initial responsive voltage” is enhanced by both the presence and conductivity of the hairs. The reason is that the responsiveness of the voltage to the odor is enhanced by the presence of the conductive hairs is that heat is transmitted through the hairs.

**Figure 23:**
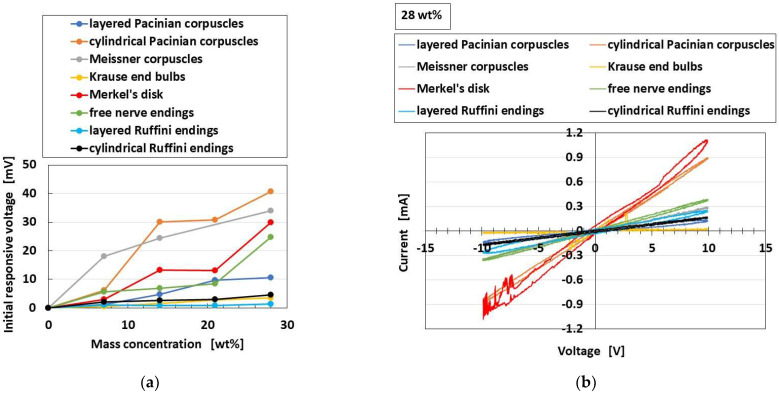
Comparison of responsive voltage and the olfactory relationship to the odor among overall receptors in the case with conductive hairs: (**a**) initial responsive voltage; (**b**) olfactory relationship.

Figure 23a shows the comparison of responsive voltage to the odor among overall receptors, in the presence of conductive hairs. In contrast, Figure 23b shows the olfactory relationship between *I* and *V* by potentiostat (HA-151B). In the case of the relation between *I* and *V*, larger current and larger area of the loop between *I* and *V* denote more response to the odor, in the presence of hairs. From both the initial responsive voltage and the olfactory relationship, cylindrical Pacinian corpuscles and Meissner corpuscles are most responsive, followed by Merkel’s disk.

### 4.7. Gustatory Response

The bare receptor is inserted into a liquid in a container, and the exemplary built-in voltage changes in the overall receptors are the same as in the case of gustatory response presented in the previous section. Therefore, the same “initial responsive voltage” was arranged.

**Figure 24:**
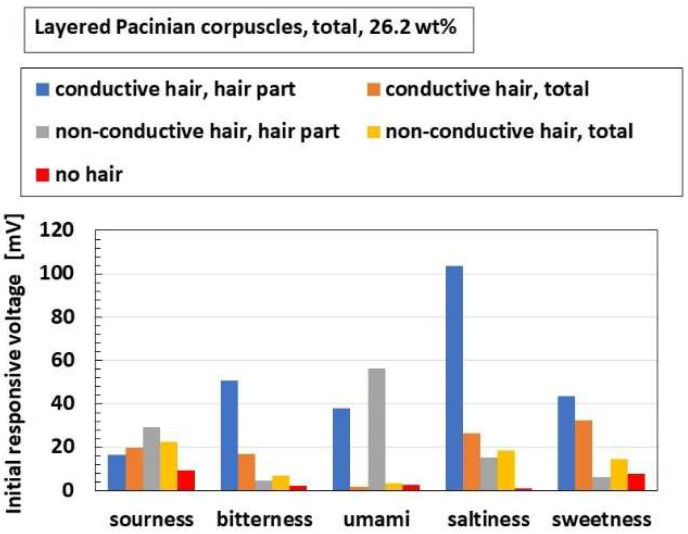
Effects of the presence and conductivity of the hairs on the gustatory response as for initial responsive voltage.

**Figure 25:**
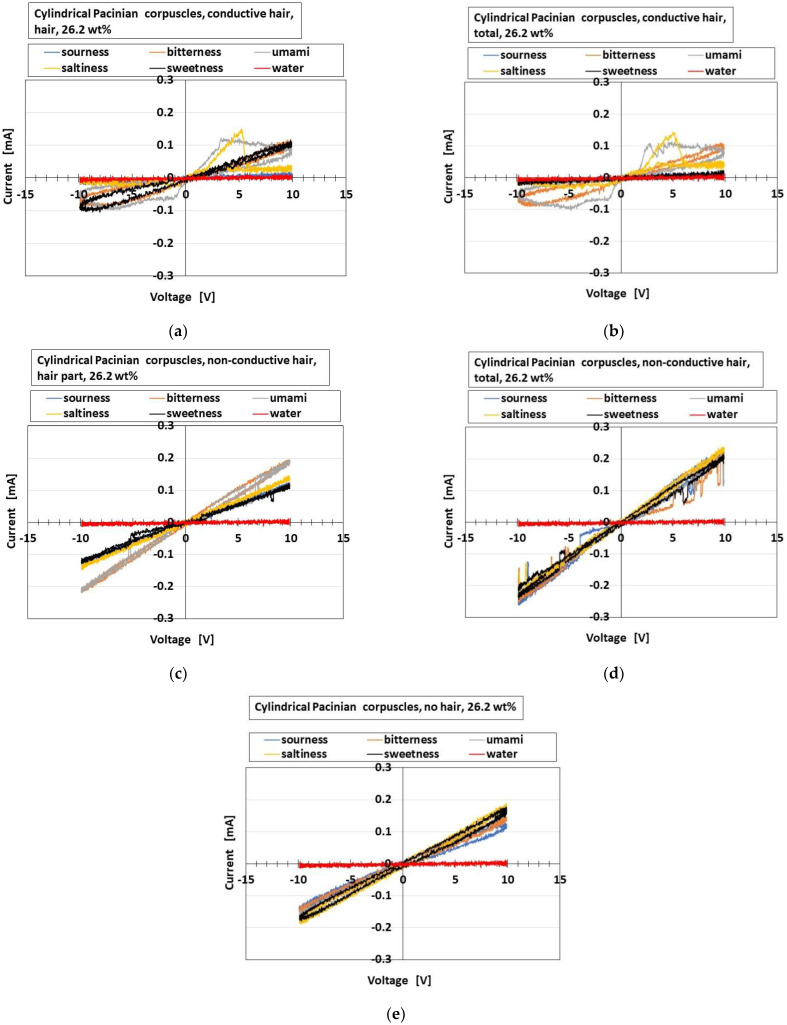
Effects of the presence and conductivity of the hairs on the gustatory response as for the gustatory relationship: (**a**) conductive hair, just hair part; (**b**) conductive hair, total hair and body parts; (**c**) nonconductive hair, just hair part; (**d**) nonconductive hair, total hair and body parts; (**e**) without hairs.

Figure 24 shows the effects of the presence and conductivity of the hairs on the parameters. Figure 25 shows the effects on the olfactory relationship between *I* and *V* by potentiostat (HA-151B). The overall receptors have the same results. In case of the relationship between *I* and *V*, the larger current and larger area of the loop between *I* and *V* denote more response to the taste. In the figures, the delineated “hair part” means to insert just the part with hairs into the liquid, and “total” means to insert all the parts of hairs and body. The responsiveness of “initial responsive voltage” is enhanced by both the hair part and the conductivity of the hairs. On the other hand, the gustatory relation is responsive according to the kind of liquids in the case of just the hair part. As a result, just the hair suffices the olfactory responsiveness. The result realizes the actual olfactory responsiveness by hairs in a nose.

**Figure 26:**
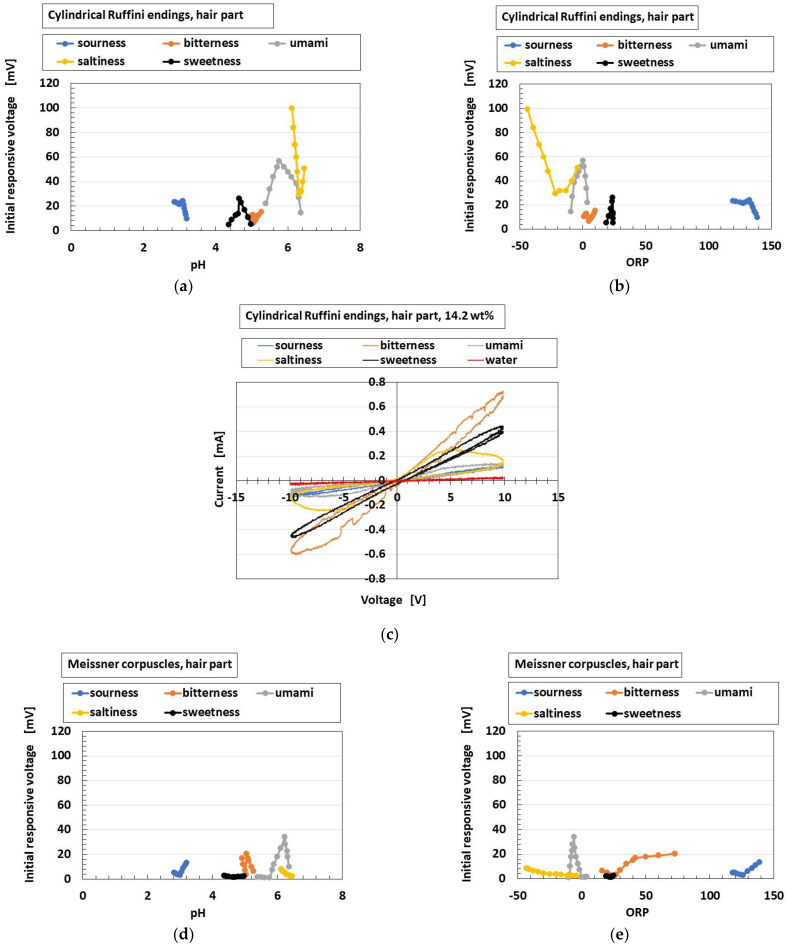

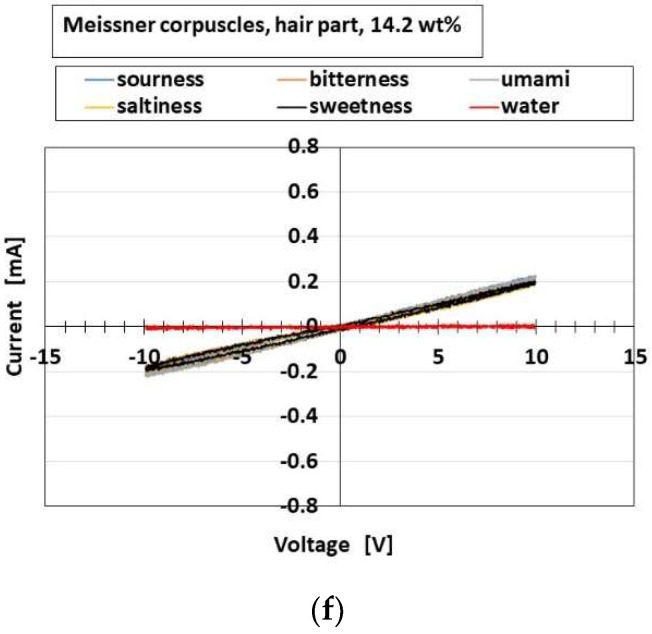
Typical results of responsive voltage to the liquids among overall receptors in cases with conductive hairs and “hair part”: (**a**–**c**) the most responsive; (**d**–**f**) not responsive; (**a**,**d**) to pH; (**b**,**e**) to ORP; (**c**,**f**) gustatory relationship.

Figure 26 shows a comparison of responsive voltage to the liquids among the overall receptors in cases with conductive hairs and “hair part”. Figure 26a–c show the most response, while Figure 26d–f show less response; the presentation here is limited because there are many results. Larger changes of initial responsive voltage in the abscissa denote more response to the taste. The results show that the cylindrical Ruffini endings are most responsive to the liquids, followed by layered Pacinian corpuscles and Merkel’s disk.

### 4.8. Consequence

**Figure A3:**
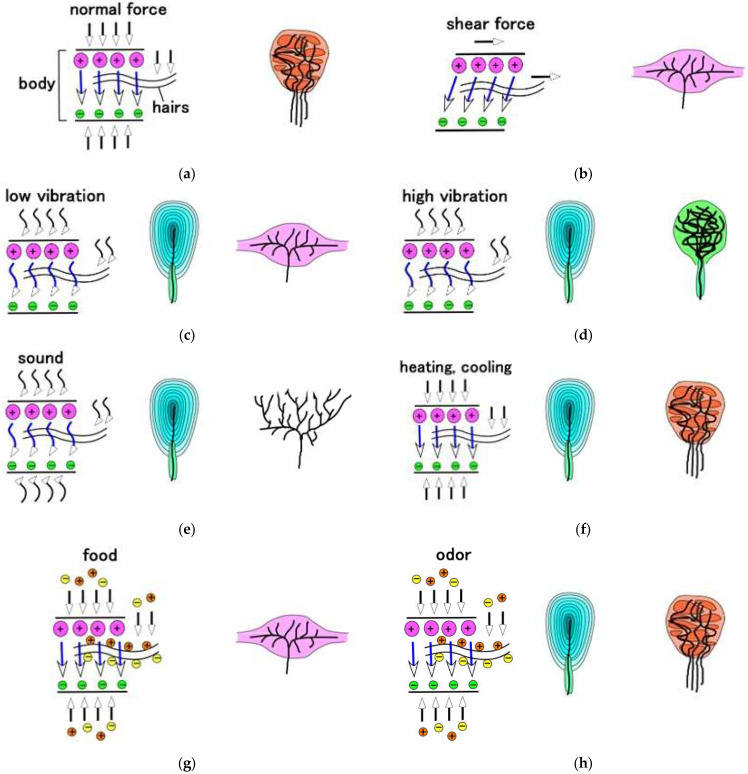
Optimum structure of the receptors corresponding to the physical model of the motion: (**a**) at normal force; (**b**) at shear force; (**c**) at vibration with low frequency; (**d**) at vibration with high frequency; (**e**) at audibility; (**f**) at thermal sensation; (**g**) at gustation; (**h**) at olfaction.

By estimating the experimental data in the foregoing sections as an optimum receptor to the extraneous stimuli, we conclusively assessed the characteristics summarized in Table 2. In the table, the double circle presents the most optimal case and the single circle the secondary optimal case. By comparing them, the tried and true results induced by many biological and mechanical investigations so far [40,41,42,43] are simultaneously shown in parenthesies indicating the general state of knowledge, highlighting in yellow the most typical findings; they are approximately in line with our obtained results. From the obtained summarized results, we can realize the characterized receptor to response by the mechanical force, vibration, thermal, auditory, gustatory, and olfactory sensations. The optimum structure is different according to the extraneous stimuli. This is illustrated with the corresponding physical model of the motion of the receptor as shown in Figure A3 in Appendix C. The physical mechanism of stimulus to the gustatory and olfactory sensations is dependent on the chemical response to the extraneous ions of food or odor, which is different from the others. As for the mechanical force and vibration, the stimulus is dependent on the deformation of the distance between the ions or molecules based on piezo-electricity and tribo-electricity as shown in Figure 7. As for thermal sensation, the stimulus is dependent on the thermal flux transferred among the particles, ions, etc.

The effect of the hairs attached on the receptors varies for each receptor depending on the presence and conductivity of the hair. The cause is presumed to be as follows. As the extraneous ions around the hairs charge electrically to the hairs, the potential between the condenser increases or decreases according to the ionized condition, which has been presented in detail by the previous study [45].

Our results reached by principles of morphological design can ultimately predict the optimal best structure of the five senses; therefore, it can be expected that more effective information on the morphological design can be obtained throughout the present experimental procedure and results. Furthermore, the coupling relationship between these morphological fabrications could be obtained. On the other hand, another approach which can ensure the acquisition of the coupling relationship is to investigate the AC electric property of the receptors. For example, the response time of stimulus can be guessed by the impedance and reactance of the receptor. Since more explanatory space is required, the detailed results will be presented in other papers.

## 5. Conclusions

To mimic the mechanoreceptors and sensory receptor cells in human skin, ear, tongue, and nose, we utilized the electrolytic polymerization technique with a magnetic-responsive fluid (HF) to fabricate receptors with the functions of tactile, auditory, gustatory, and olfactory sensations. The fabricated receptors mimicked the actual receptors with two elements, hairs and body. We detected the optimal morphological configuration of mimicked mechanoreceptors on four senses. Taking their electric circuits into account, the production of the fabricated receptors is comprehensive in providing the configuration of the real receptors. The electric circuit provides a morphological fabrication of the artificial receptor; various types of electric circuits are used to reproduce the different morphological types of receptors.

The hairs embedded in the receptors vary in function depending on their presence and conductivity. We estimated the optimum characteristics of tactile, auditory, gustatory, and olfactory experimental data for high responsivity to a range of stimuli by comparison with the results of actual cutaneous receptors and the four senses induced by the previous biological and mechanical investigations. We conclusively assessed the optimum characteristics feasible for the actual receptors of the four senses.

Contrary to the conventional fabrication technique proposed by past investigations, our technique offers a solution for artificial bioinspired sensory systems. Our proposed state-of-the-art fabrication by biomimicry using electrolytic polymerization could lead to bioinspired technology of electronic systems mimicking the five human senses and advance the creation of artificial organs and humanoid robots with high-performance sensors. This would result in revolutionary progress of sensors imitating the five senses. Our technique primarily simulates the structure of receptors with electrical response. Moreover, we can advance the morphological techniques by incorporating chemical responses. We plan to present electrolytically polymerized fabrication of sensors that mimic visual receptors in future papers.

## Figures and Tables

**Table 1:** Ingredients of HF rubber for the fabrication of the receptors.

Ingredients		HF Rubber 1	HF Rubber 2	HF Rubber 3	HF Rubber 4
	water	3 g	3 g	1 g	1 g
	sodium tungstate (VI) dehydrate (Na_2_WO_4_ 2H_2_O, Fujifilm Wako Chemical Co., Ltd., Osaka, Japan)	0.5 g	0.5 g	-	0.5 g
	TiO_2_	0.5 g	0.5 g	0.5 g	0.5 g
	HF	1 g	1 g	1 g	1 g
	NR-latex (Ulacol; Rejitex Co., Ltd., Atsugi, Japan)	3 g	3 g	3 g	3 g
	CR-latex (671A; Showa Denko Co., Ltd., Tokyo, Japan)	3 g	3 g	3 g	3 g
	carbonyl Ni powder	3 g	3 g	3 g	3 g

**Table 2:** Summary of characteristics of receptors fabricated using HF rubber.

Sensation		Response	Receptor							
			Layered Pacinian Corpuscles	Cylindrical Pacinian Corpuscles	Meissner Corpuscles	Krause End Bulbs	Merkel’s Disk	Free Nerve Endings	Layered Ruffini Endings	Cylindrical Ruffini Endings
force *^1^	normal force *^2^	mechanical	(pressure)	(pressure)	⊚	◯ (pressure)	(indentation depth by pressing, sustained touch, pressure)	(touch)	(deep pressure)	(deep pressure)
	shear force *^3^				(dynamic deformation by shearing, changes in texture)	◯	◯ (texture as fine touch)	(superficial touch)	⊚ (stretch by skin)	(stretch by skin)
vibration	low frequency		(70–1000 Hz)	⊚ (70–1000 Hz)	★ (slow vibration, 10–200 Hz)		◯, ★ (0.4–100 Hz)	★	★ (0.4–100 Hz)	⊚ (0.4–100 Hz)
	high frequency		★ (high vibration)	⊚, ★ (high vibration)		⊚ (high vibration)	★		◯	◯
auditory			⊚		◯	◯	◯	⊚ (hair)		
temperature	cold	electric		⊚	⊚	(cold)	◯	(cold)		
	hot			⊚	⊚		◯	(heat)	(warmth)	(warmth)
taste		chemical	◯			(mouth)	◯ (tongue)			⊚
smell				⊚	⊚		◯	◯		

*^1^ This category includes force conditions: touch and pressure. *^2^ This category includes force conditions: touch, low or high pressure, texture at touching, and sustained touch. *^3^ This category includes force conditions: dynamic deformation, texture at shearing, and stretch of substances. ⊚ This symbol indicates the first response. ◯ This symbol indicates the response following ⊚. ★ This symbol indicates the first response for receptors embedded in a U-rubber settled in the atmosphere. Highlighting in yellow presents the most typical findings by other study.

## Data Availability

Not applicable.

## Appendix A

Figure A1 shows the production processes of layered Pacinian corpuscles, cylindrical Pacinian corpuscles, layered Ruffini corpuscles, cylindrical Ruffini corpuscles, free nerve endings, Merkel’s disk, and Krause end bulbs. In the case of Merkel’s disk, the hairs can be adhered on the U-rubber of the finger by the Al_2_O_3_ powder attached on the tips of the hairs, as shown in Figure A1f.

## Appendix B

Experimental apparatus for mechanical, thermal, vibration, gustatory, and olfactory senses as shown in Figure A2, whose detail has been presented in our previous studies [28,31,44,45] and which adds the instruments for measuring olfactory response.

## Appendix C

The optimum structure for each external sensory response is illustrated with the corresponding physical model of the motion of the receptor as shown in Figure A3.
